# A human circulating immune cell landscape in aging and COVID-19

**DOI:** 10.1007/s13238-020-00762-2

**Published:** 2020-08-11

**Authors:** Yingfeng Zheng, Xiuxing Liu, Wenqing Le, Lihui Xie, He Li, Wen Wen, Si Wang, Shuai Ma, Zhaohao Huang, Jinguo Ye, Wen Shi, Yanxia Ye, Zunpeng Liu, Moshi Song, Weiqi Zhang, Jing-Dong J Han, Juan Carlos Izpisua Belmonte, Chuanle Xiao, Jing Qu, Hongyang Wang, Guang-Hui Liu, Wenru Su

**Affiliations:** 1 State Key Laboratory of Ophthalmology, Zhongshan Ophthalmic Center, Sun Yat-sen University, 510060 Guangzhou, China; 2 State Key Laboratory of Membrane Biology, Institute of Zoology, Chinese Academy of Sciences, 100101 Beijing, China; 3 National Center for Liver Cancer, Second Military Medical University, 200433 Shanghai, China; 4 Department of Critical Care, Wuhan Hankou Hospital, 430012 Wuhan, China; 5 State Key Laboratory of Stem Cell and Reproductive Biology, Institute of Zoology, Chinese Academy of Sciences, 100101 Beijing, China; 6 Institute for Stem Cell and Regeneration, CAS, 100101 Beijing, China; 7 University of Chinese Academy of Sciences, 100049 Beijing, China; 8 Advanced Innovation Center for Human Brain Protection, National Clinical Research Center for Geriatric Disorders, Xuanwu Hospital Capital Medical University, 100053 Beijing, China; 9 CAS Key Laboratory of Genomic and Precision Medicine, Beijing Institute of Genomics, Chinese Academy of Sciences, 100101 Beijing, China; 10 China National Center for Bioinformation, 100101 Beijing, China; 11 Peking-Tsinghua Center for Life Sciences, Academy for Advanced Interdisciplinary Studies, Center for Quantitative Biology (CQB), Peking University, 100871 Beijing, China; 12 Gene Expression Laboratory, Salk Institute for Biological Studies, La Jolla, CA, USA

**Keywords:** aging, single-cell sequencing, blood, COVID-19, immune cells

## Abstract

Age-associated changes in immune cells have been linked to an increased risk for infection. However, a global and detailed characterization of the changes that human circulating immune cells undergo with age is lacking. Here, we combined scRNA-seq, mass cytometry and scATAC-seq to compare immune cell types in peripheral blood collected from young and old subjects and patients with COVID-19. We found that the immune cell landscape was reprogrammed with age and was characterized by T cell polarization from naive and memory cells to effector, cytotoxic, exhausted and regulatory cells, along with increased late natural killer cells, age-associated B cells, inflammatory monocytes and age-associated dendritic cells. In addition, the expression of genes, which were implicated in coronavirus susceptibility, was upregulated in a cell subtype-specific manner with age. Notably, COVID-19 promoted age-induced immune cell polarization and gene expression related to inflammation and cellular senescence. Therefore, these findings suggest that a dysregulated immune system and increased gene expression associated with SARS-CoV-2 susceptibility may at least partially account for COVID-19 vulnerability in the elderly.

## Introduction

The world population is undergoing a rapid expansion of older adults, and thus, exploring how to stay healthy with age has become an urgent global focus. Aging leads to numerous physiological changes, including the deterioration of the immune system, rendering the elderly more susceptible to infections, such as the COVID-19 pandemic, and poor responses to vaccines (Ciabattini et al., 2018; Alpert et al., 2019; Onder et al., 2020; Verity et al., 2020). Changes observed during aging are often reflected as alterations in the composition and functional declines of diverse immune cells. For T cells (TCs), the high frequency of naive cells in young humans progressively decreases along with the accumulation of highly differentiated memory cells (Hakim and Gress, 2007), whereas nonclassical monocytes (MCs) with high levels of plasma tumor necrosis factor (TNF)-α and interleukin (IL)-8 accumulate with age (Ong et al., 2018). In addition, senescence of the immune system in the elderly has been termed “inflammaging”, which refers to increased levels of tissue and circulating proinflammatory cytokines in the absence of an immunological threat (Panda et al., 2009; Franceschi et al., 2018). Overall, aging is associated with changes in the structure of diverse immune compartments, where accumulating dysfunctional subsets contribute to immune failure.

Seminal studies have provided insights into the compositions and functional alterations occurring during aging, primarily based on previously described markers detected in pooled heterogeneous cell populations. The recent development of unbiased high-throughput single-cell technologies with high accuracy and specificity has begun to change immunological studies, as researchers worldwide are ushering in the new field of systems immunology. By using single-cell sequencing, recent studies have reported that cell-to-cell transcriptional variability increases with age in CD4^+^ TCs (Bahar et al., 2006; Martinez-Jimenez et al., 2017) and in leukocytes from old mouse lungs (Angelidis et al., 2019). Aging also increases the variations in chromatin modifications of human immune cells (Cheung et al., 2018). Very recently, many immunological phenotypes, such as intratissue accumulation of proinflammatory cells, have been reported in aging rodent and primate models (Messaoudi et al., 2006; Watson et al., 2017; Hammond et al., 2019; Ma et al., 2020). However, a comprehensive aging cell atlas of human peripheral blood that systematically connects all the blood lineages and cell subtypes has not yet been constructed.

Here, we applied single-cell RNA sequencing (scRNA-seq), mass cytometry (CyTOF), and single-cell assay for transposase-accessible chromatin sequencing (scATAC-seq) to comprehensively characterize the properties of peripheral blood mononuclear cells (PBMCs) in young and old adults. We also enrolled young and aged COVID-19 patients in the incipient stage and recovery stage to explore how age influenced the capacity for recovery and prognosis of COVID-19 infection and to better understand the influence of immune dysregulation in aging and infection. Our data revealed that aging promotes the polarization of TCs from naive and memory to effector, exhausted and regulatory subtypes and increases the numbers of late natural killer cells (NKs), age-associated B cells (BCs), inflammatory MCs, and dysfunctional dendritic cells (DCs). With single-cell paired T/B cell receptor sequencing (scTCR/BCR-seq), we uncovered decreased diversity and increased clonality of effector, cytotoxic and exhausted CD8^+^ TC subsets in TCs and age-associated B subsets in BCs with age. Notably, aging increased the expression of inflammation-related genes, senescence-related genes, and coronavirus susceptibility genes in specific cell subtypes. Most impressively, COVID-19 caused similar immune cell landscape changes to that of aging and further increased aging-induced immune cell polarization and upregulation of inflammatory genes. Increased SARS-CoV-2 susceptibility gene expression and inflammatory MCs and decreased TCs aggravate inflammatory storms and lymphopenia (Mehta et al., 2020; Merad and Martin, 2020; Zhou et al., 2020) and likely underlie the high susceptibility and mortality of old patients. Overall, this work expands our knowledge of aging via single-cell transcriptomic, proteomic and chromatin accessibility immune cell profiling and highlights critical nodes between the dysregulated immune system and infections that may serve to modulate the process of inflammaging.

## Results

### Cohort characteristics and single-cell analysis of PBMCs in young and aged adults

**Figure 1:**
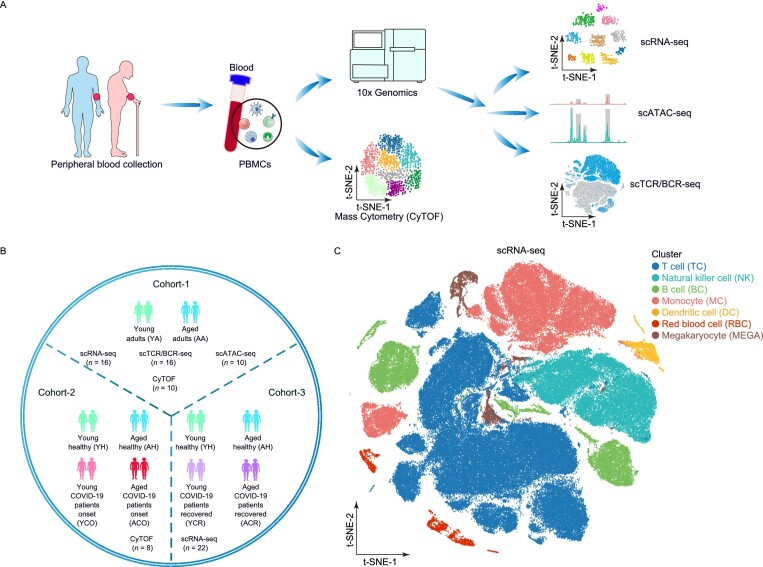

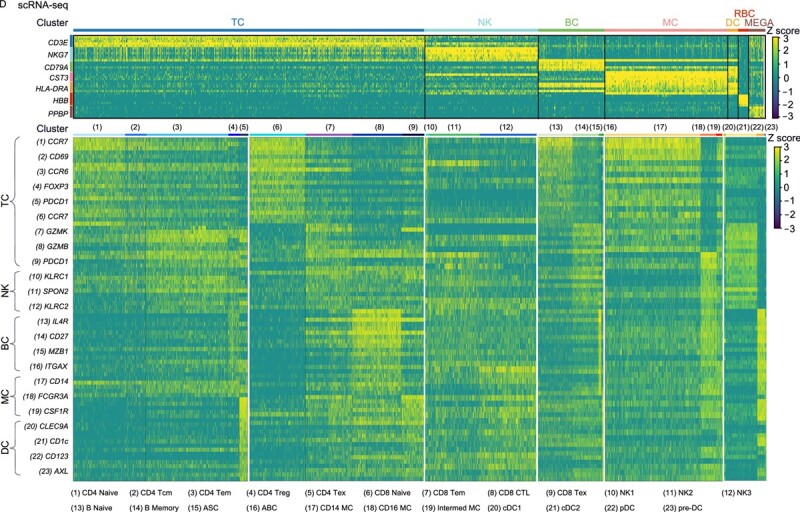
**Schematic illustration of the collection and data processing of PBMC from young and aged group**. (A) Flowchart overview of PBMC collection in young and aged adults followed by scRNA-seq, mass cytometry, scATAC-seq and scTCR/BCR-seq experiments. (B) Schematic illustration of experimental cohorts; cohort-1: young and aged adults, cohort-2: young and aged healthy individuals, young and aged adults with COVID-19 onset, cohort-3: young and aged healthy individuals, young and aged adults recovered from COVID-19, matched with analysis as indicated: single-cell proteomic data from CyTOF studies, gene expression data from scRNA-seq studies, chromosomal accessibility data from scATAC-seq, and TCR and BCR repertoire data from scTCR/BCR-seq. (C) t-SNE projections of PBMCs derived from scRNA-seq data in cohort-1. (D) Heatmaps showing scaled expression of discriminative gene sets for each cell type and cell subset. Color scheme is based on z-score distribution from −3 (purple) to 3 (yellow)

To generate a comprehensive immune cell atlas reflecting cellular and systemic adaptations resulting from age and/or COVID-19 infection, we integrated scRNA-seq, CyTOF, scATAC-seq and scTCR/BCR-seq of single-cell PBMC suspensions collected from 3 separate cohorts (Fig. 1A, 1B, and Table S1A–G). In cohort-1, comprising young healthy adults (YA) (20–45 years old) and aged healthy adults (AA) (≥60 years old), we combined CyTOF (*n* = 10) and scATAC-seq (*n* = 10) with scRNA-seq (*n* = 16) and scTCR/BCR-seq (*n* = 16); in cohort-2, comprising young healthy (YH) individuals (30–45 years old), aged healthy (AH) individuals (≥60 years old), young COVID-19 onset patients (YCO) (30–50 years old) and aged COVID-19 onset patients (ACO) (≥70 years old), we performed CyTOF analysis (*n* = 8); and in cohort-3, comprising YH individuals, AH individuals, young recovered COVID-19 patients (YCR) (30–50 years old) and aged recovered COVID-19 patients (ACR) (≥70 years old), we performed scRNA-seq (*n* = 22) (Fig. 1B). By combining scRNA-seq, CyTOF, scATAC-seq and scTCR/BCR-seq analysis, we created a comparative framework detailing the impact of aging on cell type distribution and immune cell functions at the transcriptional, proteomic, and chromatin accessibility levels in cohort-1. In cohort-2, we measured single-cell protein expression using a 26-marker CyTOF panel to discover early cellular changes in incipient COVID-19 patients and how those changes were affected by age. Finally, in cohort-3, we compared cellular differences between young and aged recovered COVID-19 patients by scRNA-seq analysis (Fig. 1B).

We analyzed PBMC single-cell suspensions by CyTOF for the protein expression of several lineage-, activation- and trafficking-associated markers and converted them to barcoded scRNA-seq libraries using 10x Genomics for downstream scRNA-seq, scATAC-seq and scTCR/BCR-seq analysis. CellRanger software and the Seurat package were used for initial processing of the sequencing data. Quality metrics included numbers of unique molecular identifiers (UMIs), genes detected per cell, and reads aligned that were comparable across different research subjects. We identified red blood cells (RBCs), megakaryocytes (MEGAs) and five major immune cell lineages (TCs, NKs, BCs, MCs and DCs) based on the expression of canonical lineage markers and other genes specifically upregulated in each cluster (Figs. 1C, 1D and S1A–C). In accordance with the scRNA-seq results, we identified five immune cell lineages (TCs, NKs, BCs, MCs and DCs) in CyTOF using t-distributed stochastic neighbor embedding (t-SNE), an unbiased dimensionality reduction algorithm (See Table S2 for a list of antibodies) (Fig. S2A–D). Cell-type-specific marker genes were determined by differential gene expression values between clusters positioned and visualized in a t-SNE plot (Figs. S1 and S2). The definition of cell types in clusters in the t-SNE maps was comparable between old and young individuals (Figs. S1B and 2B) both by scRNA-seq and CyTOF, indicating that the cell type identity was not altered with age.

**Figure 2:**
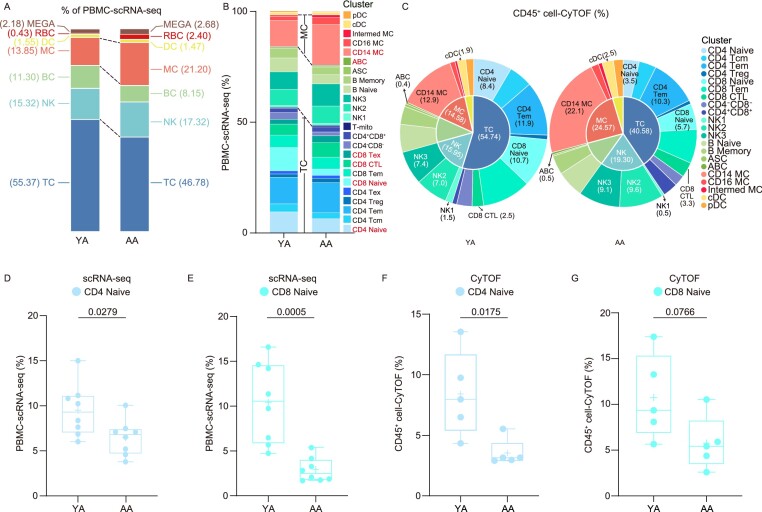

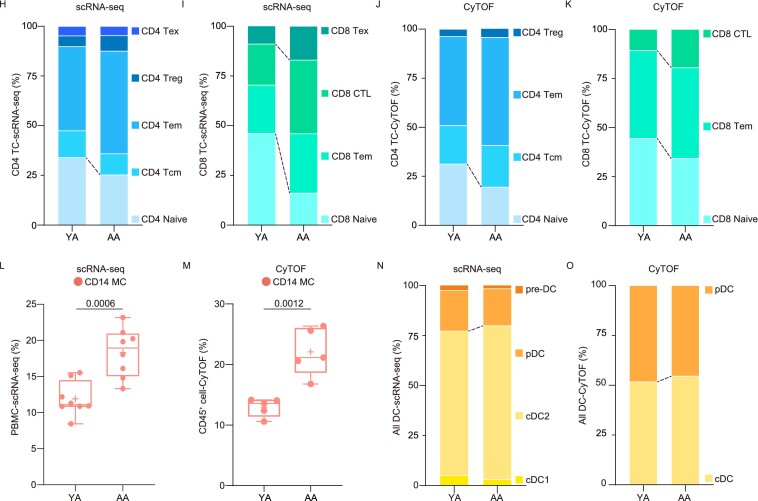
**Changes in cell proportions during aging**. (A) Bar chart of the relative percentage of immune cell types derived from scRNA-seq data in PBMCs. (B) Bar chart of the relative percentage of immune cell subsets derived from scRNA-seq data in PBMCs. The focused cell-subsets have been marked red. (C) Pie charts showing relative cluster abundances derived from mass cytometry data in the YA and AA groups. (D) Percentage of CD4 Naive cells in PBMCs from YA (*n* = 8) and AA (*n* = 8) groups. (E) Percentage of CD8 Naive cells in PBMCs from YA (*n* = 8) and AA (*n* = 8) groups. (F) Percentage of CD4 Naive cells in CD45^+^ cells from YA (*n* = 5) and AA (*n* = 5) groups. (G) Percentage of CD8 Naive cells in CD45^+^ cells from YA (*n* = 5) and AA (*n* = 5) groups. (H) Bar chart of the relative percentage of CD4^+^ T cell subsets derived from scRNA-seq data in PBMCs. (I) Bar chart of the relative percentage of CD8^+^ T cell subsets derived from scRNA-seq data in PBMCs. (J) Bar chart of the relative percentage of CD4^+^ T cell subsets derived from mass cytometry data in CD45^+^ cells. (K) Bar chart of the relative percentage of CD8^+^ T cell subsets derived from mass cytometry data in CD45^+^ cells. (L) Percentage of CD14 monocytes in PBMCs from YA (*n* = 8) and AA (*n* = 8) groups. (M) Percentage of CD14 monocytes in CD45^+^ cells from YA (*n* = 5) and AA (*n* = 5) groups. (N) Bar chart of the relative percentage of DC subsets derived from scRNA-seq data in PBMCs. (O) Bar chart of the relative percentage of DC subsets derived from mass cytometry data in CD45^+^ cells. *P* values are based on two-tailed Mann-Whitney-Wilcoxon tests between groups

### Dissection of immune cell subtypes in the cellular aging ecosystem

To classify each cell subpopulation in an unbiased manner, we separately reclustered the cells of each lineage. By analyzing the most significantly upregulated genes in each cluster in scRNA-seq analysis, we identified five distinct subsets of CD3^+^ TCs (Fig. S3A), five distinct subsets of CD4^+^ TCs (Fig. S3B), four distinct subsets of CD8^+^ TCs (Fig. S3C), three distinct subsets of NKs (Fig. S3D), four distinct subsets of BCs (Fig. S3E), three distinct subsets of MCs (Fig. S3F) and four distinct subsets of DCs (Fig. S3G, see Table S3A for the details).

Aging affects the development and function of TCs and NKs (Pinti et al., 2016). We identified known T cell subsets, including *CD4*^+^, *CD8*^+^, *CD4*^+^*CD8*^+^, *CD4*^−^*CD8*^−^ and proliferative T cells (mitotic T cells, T-mito), based on the expression of canonical lineage markers (Fig. S3H). The CD4^+^ T cells were subdivided into five classes: *CCR7*^high^*CD69*^low^ naive CD4^+^ T cells (CD4 Naive); *CCR7*^med^*CD69*^high^*CCR6*^−^ central memory CD4^+^ T cells (CD4 Tcm); *CCR6*^+^ effector memory CD4^+^ T cells (CD4 Tem); *FOXP3*^+^ regulatory T cells (CD4 Treg) and *PDCD1*^+^ exhausted CD4^+^ T cells (CD4 Tex) (Fig. S3I). The CD8^+^ T cells were subdivided into four classes: *CCR7*^+^ naive CD8^+^ T cells (CD8 Naive); *GZMK*^+^ effector memory CD8^+^ T cells (CD8 Tem); *GZMB*^+^*GNLY*^+^ cytotoxic CD8^+^ TCs (CD8 CTL) and *PDCD1*^+^ exhausted CD8^+^ T cells (CD8 Tex) (Fig. S3J).

Analysis of NK cell-status identified circulating NKs with three separate immune states (Fig. S 3D): the *CD16* (*FCGR3A*)^low^*CD56* (*NCAM1*)^bright^ NK population (NK1), the *CD16*^high^*CD56*^dim^*CD57* (*B3GAT1*)^−^ low-cytotoxic NK compartment (NK2) and the *CD16*^high^*CD56*^dim^*CD57*^+^ late NK population (NK3) (Fig. S3K). In addition, we identified four major peripheral B cell subsets: *IL4R*^+^*IGHD*^+^ naive B cells (Naive BCs); *CD27*^+^*IGHG1*^+^ memory B cells (Memory BCs); plasma cells or so-called antibody-secreting cells (ASCs), expressing high level of immunoglobulin genes *MZB1*; and a subset of *ITGAX*^+^ B cells defined as age-associated B cells (ABCs) (Fig. S3L).

In human peripheral blood myeloid cells (including MCs and DCs), known to promote antigen presentation and inflammatory activities, we identified seven transcriptionally distinct subsets: *CD14*^high^*CD16*^−^ classical monocytes (CD14 MCs), *CD14*^+/−^*CD16*^high^ nonclassical monocytes (CD16 MCs), *CD14*^+^*CD16*^+/−^ intermediate monocytes (Intermed MCs) (Fig. S3M), *CLEC9A*^+^ conventional DC1 (cDC1), *CD1c*^+^ cDC2 conventional DC2 (cDC2), *CD123* (*IL3RA*)^+^*CLEC4C*^*+*^ plasmacytoid DCs (pDCs) (Fig. S3N), and dendritic cell precursors (pre-DCs) expressing *AXL* and *CD123* (Grabiec and Hussell, 2016; Ruffin et al., 2019) (Fig. S3N). Therefore, we targeted the immune cell changes based on more precise classification of each subgroup.

To further verify the aging-associated change in the cell ratio, we performed single-cell analysis at the protein level. Similar to the cell clusters and subsets in scRNA-seq results, in CyTOF analysis, we identified 21 sub-clusters with nine subsets of TCs (CD4 Naive, CD4 Tcm, CD4 Tem, CD4 Treg, CD8 Naive, CD8 Tem, CD8 CTL, CD4^+^ CD8^+^, CD4^−^ CD8^−^), three subsets of NKs (CD56^bright^ NK1, CD16^+^CD57^−^ NK2 and CD16^+^CD57^+^ NK3), four subsets of BCs (Naive BC, Memory BC, ASCs, and ABCs), three subsets of MCs (CD14^high^ MCs, CD16^high^ MCs and intermediate MCs), and two subsets of DCs (pDCs and cDCs) (Fig. S4A–K, see Table S3B for the details).

### Aging shifts the cellular composition toward extreme effector phenotypes

To delineate how cell-type composition changed with aging, we separately compared the proportions of each cell type across major cell types between the YA and AA groups. We observed changes at the single-cell transcriptional level, which were further confirmed at the protein level by CyTOF. Globally, we found that TCs and BCs, especially the former, decreased by approximately 10% in all PBMCs with scRNA-seq analysis (Fig. 2A, 2B, and S5A) and by 15% with CyTOF (Figs. 2C and S5B). In contrast, MCs increased by approximately 7% in scRNA-seq analysis (Figs. 2A, 2B, and S5A) and by 10% in CyTOF (Figs. 2C and S5B).

The composition of cell subsets across all cell lineages differed between the YA and AA groups. Among TCs, CD4^+^ TCs were increased, CD8^+^ TCs were decreased, and CD4^+^CD8^+^ and proliferating T cells were increased in the AA group (Fig. 2B and 2C). Moreover, naive TCs, especially CD4 Naive and CD8 Naive, showed a common distribution in the YA group but were reduced in the aged group (*P* = 0.0175, Fig. 2D–G). Conversely, effector, memory and exhausted cell subsets were dominant in the aged group (Fig. 2H–K). The AA group also had a diminished proportion of the CD56^bright^ NK1 population and an expansion of the NK2 and late NK3 populations (Fig. S5C and S5D). Analysis of BC clusters revealed that Naive BCs were decreased while ABCs were mildly increased in the AA group compared to the YA group (Fig. S5E and S5F).

Our data also showed that elderly research subjects had increased MC subsets, particularly classical CD14 MCs and, to some extent, nonclassical CD16 MCs and intermediate MCs (Figs. 2L, 2M, S5G, and S5H). Overall MC growth mainly resulted from CD14 MC enrichment (*P* = 0.0012, Fig. 2M). However, given that CD14 MCs made up 70%–80% of the MCs population, the increase we observed in CD16 MCs was more remarkable as a change in the overall population proportion between the AA and YA groups, which was not observed for intermediate MCs between these groups (Fig. S5G–J). A similar analysis of the DC subset composition showed that the percentage of cDC2 cells increased, whereas cDC1, pDC, and pre-DC decreased with age (Figs. 2N, 2O, and S5I).

In summary, these results demonstrate that aging induces an immune dysfunction shift into effector and inflammatory cell populations.

### Identification of aging-related cell-type-specific transcriptional expression changes

**Figure 3:**
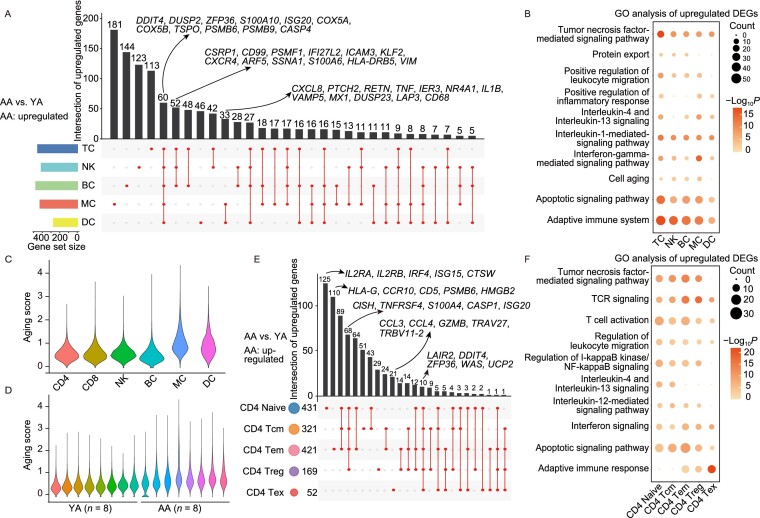

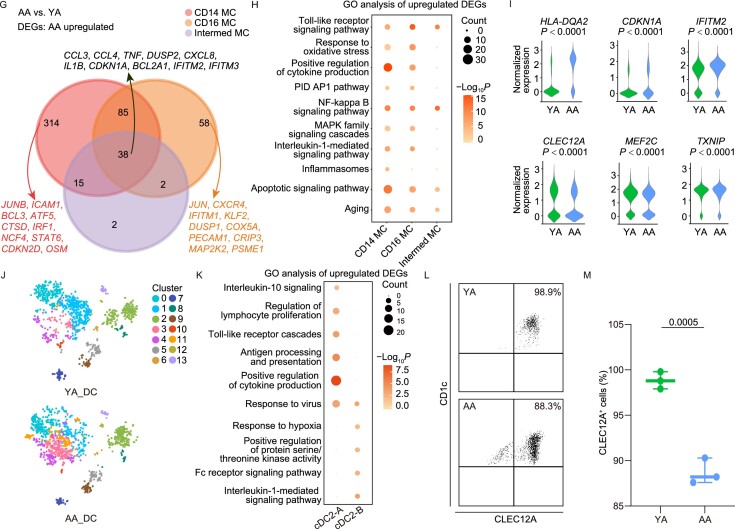
**Changes in transcriptional profiles during aging**. (A) UpSet Plot showing the integrated comparative analysis of upregulated DEGs in major immune cell lineages between YA and AA groups. Upregulated DEGs: upregulated in AA, downregulated in YA group. (B) Representative GO terms and pathways enriched in upregulated DEGs based on functional enrichment analysis in major immune cell populations. *P* value was derived by a hypergeometric test. (C) Distribution and comparison of the aging score in immune cell populations. (D) Distribution and comparison of the aging score in all cells of each sample. (E) UpSet plot showing the integrated comparative analysis of upregulated DEGs in CD4^+^ T cells between YA and AA groups. Upregulated DEGs: upregulated in AA, downregulated in YA. The count showing the number of DEGs. (F) Representative GO terms and pathways enriched in upregulated DEGs based on functional enrichment analysis in CD4^+^ T cells. *P* value was derived by a hypergeometric test. (G) Venn diagram showing integrated comparative analysis of upregulated DEGs in monocytes between YA and AA groups. Upregulated DEGs: upregulated in AA, downregulated in YA. The count showing the number of DEGs. (H) Representative GO terms and pathways enriched in upregulated DEGs based on functional enrichment analysis in monocytes. *P* value was derived by a hypergeometric test. (I) Violin plots showing the distribution of normalized expression levels of selected aging-associated genes in all DC cluster between YA and AA groups. (J) t-SNE plots segregated on the basis of DC subsets. (K) Representative GO terms and pathways enriched in biased DEGs of cDC2-A and cDC2-B clusters. *P* value was derived by a hypergeometric test. (L) CLEC12A expression in cDC2 is shown as flow cytometry histogram. (M) Percentage of CLEC12A^+^ cells in cDC2. *P* value are based on two-tailed Mann-Whitney-Wilcoxon tests between YA and AA groups (*n* = 3/group)

To identify cell-subtype-specific gene signatures associated with aging, we performed an integrated comparative analysis of differentially expressed genes (DEGs) from blood immune cells in the YA and AA groups. We found that blood immune cells showed heterogeneous transcriptional changes affected by aging based on the number of DEGs. Strikingly, BC was the cell type most affected by aging, followed by TC and MC (Figs. 3A, S6A; Table S4A–E). Specifically, we found a set of 60 genes whose expression was increased in all kinds of immune cells, indicative of general oxidative stress (e.g., *DDIT4*, *CASP4*, *TSPO*) and an inflammatory state (e.g., *DUSP2*, *S100A10*, *COX5A*, *PSMB6*) across cell populations (Fig. 3A). Conversely, genes with decreased expression shared across all cell populations included *DDX17*, *RBM39*, and *SCAF11*, which are involved in RNA splicing (Fig. S6A and S6B). Consistent with our understanding of the main immune cell lineages, we found that the myeloid and lymphocyte cell lineages were characterized by unique gene expression spectra, whereas TCs showed the highest heterogeneity in DEGs. To explore the biological implications of our data in the context of aging, we used Gene Ontology (GO) and pathway analysis for each immune cell population. Common aging-upregulated biological pathways included TNF signaling, IL-1 signaling, the apoptotic signaling pathway, and the adaptive immune response (Fig. 3B). We found that these pathways were especially enhanced in TCs. In addition, aging-upregulated biological pathways in MCs were enriched for interferon-gamma (IFN-γ) signaling and cell aging (Fig. 3B). To assess the impact of aging on circulating immune cells, we selected the top 20 genes of the 60 total genes that were upregulated in all immune cells (Fig. 3A) and calculated aging scores across all immune cell types. We found that MCs and DCs had the highest scores, suggesting that senescent cells are most likely present in these cell populations (Fig. 3C). Moreover, when calculating the scores of individual samples, we found that individuals in the AA group had consistently higher scores than individuals in the YA group (Fig. 3D), suggesting that aging-score assessments are suitable for studying aging-related immune dysfunction.

By analyzing age-associated DEGs in CD4^+^ TCs, we found enrichment in inflammatory and effector genes (Tables S5A and 6A–E). To determine cell-subtype-specific gene signatures within different CD4^+^ TC subpopulations, we generated UpSet plots of upregulated DEGs in different CD4^+^ TC subsets. We found a range of subtype-specific patterns, including the *IL2 receptor* (*IL2RA*) in Naive cells, *CCR10* in Tem, and *GZMB* and *TRBV11-2* in Tex (Fig. 3E). GO and pathway analysis of the DEGs demonstrated that effector and memory subsets were most affected by aging based on the number of DEGs. For example, in CD4 Tem, TNF, interleukin signaling and apoptotic pathways were enhanced, whereas mRNA processing was impaired (Figs. 3F and S6C). Analysis of CD8^+^ TC status indicated that the AA group had increased expression of chemokines and granzyme family members (Fig. S6D; Tables S5B and 7A–D). Moreover, aging was associated with a decreased proportion of CD8 Naive with increased apoptotic signaling pathway and lymphocyte activation and an expanded CD8 Tem compartment with increased cytokine production as well as reduced chromatin remodeling and antiviral function (Fig. S6E and S6F). In addition, T-mito in aged group was associated with the upregulated inflammatory signaling molecules *HLA-DRB5*, *PDCD5* and *PSMA2* (Fig. S6G; Table S5C) and inflammatory pathways (Fig. S6H).

Analysis of NKs status revealed that the AA group had a smaller fraction of the CD56^bright^ NK1 population and expanded late low-cytotoxic NK subsets than the YA group. Notably, NKs in the AA group had increased expression of *DDIT4*, *ISG20*, and *CASP4* and decreased expression of *DDX17*, *PCBP1* and *TRIM56* (Figs. 3A, S6A; Table S8A–C). These genes were mostly enriched in apoptotic signaling pathways and cellular responses to lipopolysaccharide, along with decreased virus defense responses (Fig. S6I and S6J). As for BCs, we found increased expression of *JUNB*, *IGHA1*, *SSR4* and *CXCR4*, indicative of increased memory BC signature and activity during aging (Figs. 3A, S7A; Table S9A–D). Moreover, the comparative functional analysis of aging-associated DEGs revealed that Naive BC in the AA group had increased cytokine-mediated signaling pathways (Fig. S7B). Additionally, analysis of downregulated DEGs and pathways in the AA group demonstrated that BCs were associated with reduced viral defense responses (Fig. S7C). These results indicate that NKs and BCs lose their capacity for antiviral activity with upregulated inflammatory states in aging.

We next studied aging-associated DEGs in MCs and found enrichment in inflammatory genes, such as *IL1B*, *TNF* and *CXCL8,* in the AA group (Fig. 3A). All MC subsets had increased expression of the chemokines, *TNF*, *IL1B* and *CDKN1A* and decreased expression of *SIGLEC14* and *CLEC12A* (Figs. 3G, S6A; Table S10A–C). Analysis of aging-related DEGs demonstrated that the CD14 MC subset was most affected by aging, as reflected by the increased NOD-like receptor signaling pathway, NF-κB signaling pathway, Toll-like receptor signaling pathway, inflammasome pathway, and MAPK pathway (Fig. 3H) and the obvious decrease in RNA splicing, autophagy, and vesicle-mediated transport (Fig. S7D).

To complete our DEG and GO survey of immune lineage cells and their subtypes, we next analyzed aging-associated DEGs in DCs in the YA and AA groups (Figs. 3I, S7E and S7I; Table S11A–D). Among the upregulated DEGs, IFN-stimulated genes (*IFITM2*, *ISG20*), *TNF* and *IL1B* indicated an overactive inflammatory response in DC clusters in the AA group (Figs. 3A, 3I, S7E, and S7I). We observed that overrepresented pathways in DCs from the AA group included apoptotic, MAPK, IL-1, and IFN-γ signaling pathways (Fig. S7F). Notably, *CLEC12A* and *TXNIP*, which are critical for the antigen-presentation function of DCs; and *MALAT1* and *AHR*, which are critical to inducing tolerogenic DCs (Son et al., 2008; Hutten et al., 2016; Wu et al., 2018), were decreased in AA DCs (Figs. 3I, S7G, and S7I), reflecting the decreased antigen-presenting ability of aged DCs (Fig. S7H). These results indicate that DCs acquire an inflammatory state with age but lose the antigen-presenting ability.

Within DC clusters, we found distinct aging manifestations in the cDC2 subsets by comparing DC clusters in the t-SNE map (Fig. 3J). Cells from the YA group grouped together in clusters 0 and 1 (named cDC2-A), whereas cells in AA group grouped distinctively in clusters 3, 4, 10 and 11 (named cDC2-B). The expression signature of cDC2-A cells included antigen presentation-related genes such as *AHR*, *CLEC4E*, and *CLEC12A*, whereas the expression signature of cDC2-B cells included inflammatory and aging-associated genes such as IFN-stimulated genes, *IL1B*, *CDKN2D*, *DDIT4*, *CXCL8*, and *DUSP2* (Fig. S7J and S7K). Moreover, the comparative functional analysis of DEGs between the two clusters indicated that cDC2-A had intact immune regulation and antigen presentation function, while aging-related cDC2-B with high *HLA-DQA2* expression exhibited increased inflammatory signaling pathways, such as the response to hypoxia and IL-1 signaling (Fig. 3K). We further confirmed that CLEC12A^+^ cDC2s were decreased in aging by FACS (Fig. 3L and 3M). Taken together, these findings indicate that aging curtails DC antigen presentation ability and upregulates inflammatory and aging-associated gene expression in DCs.

### Identification of aging-related cell-type-specific chromosomal accessibility changes

**Figure 4:**
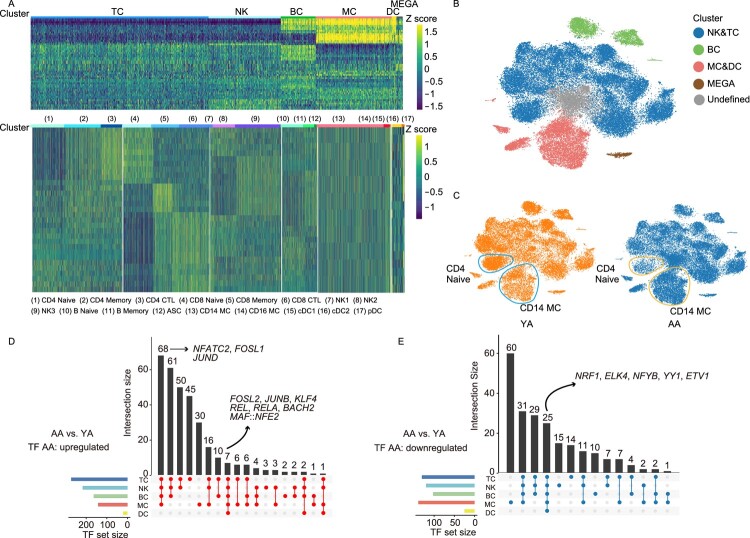

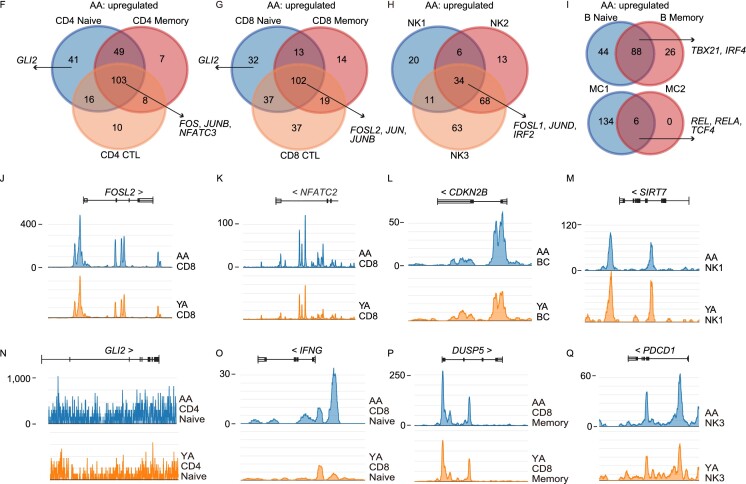
**Changes in chromosomal accessibility during aging**. (A) Heatmaps showing scaled expression of discriminative gene sets for each cell type and cell subset. Color scheme is based on z-score distribution from −1.5 (purple) to 1.5 (yellow). (B) t-SNE projections of PBMCs derived from scATAC-seq data. (C) t-SNE plots segregated by YA and AA groups. (D) UpSet plot showing the integrated comparative analysis of upregulated differentially expressed transcription factors (DETs) in major immune cell populations between YA and AA groups. Upregulated DETs: upregulated in AA, downregulated in YA. The count showing the number of DETs. (E) UpSet plot showing the integrative comparative analysis of downregulated DETs in major immune cell populations between YA and AA groups. Downregulated DETs: upregulated in YA, downregulated in AA. The count showing the number of DETs. (F) Venn diagram showing integrated comparative analysis of upregulated DETs in CD4^+^ T cells between YA and AA groups. Upregulated DETs: upregulated in AA, downregulated in YA. The count showing the number of DETs. (G) Venn diagram showing integrated comparative analysis of upregulated DETs in CD8^+^ T cells between YA and AA groups. Upregulated DETs: upregulated in AA, downregulated in YA. The count showing the number of DETs. (H) Venn diagram showing integrated comparative analysis of upregulated DETs in NK cells between YA and AA groups. Upregulated DETs: upregulated in AA, downregulated in YA. The count showing the number of DETs. (I) Venn diagram showing integrated comparative analysis of upregulated DETs in B cells (top) and monocytes (bottom) between YA and AA groups. Upregulated DETs: upregulated in AA, downregulated in YA. The count showing the number of DETs. (J) Mean scATAC-seq coverage at *FOSL2* loci in CD8^+^ T cells. (K) Mean scATAC-seq coverage at *NFATC2* loci in CD8^+^ T cells. (L) Mean scATAC-seq coverage at *CDKN2B* loci in B cells. (M) Mean scATAC-seq coverage at *SIRT7* loci in NK1 cells. (N) Mean scATAC-seq coverage at *GLI2* loci in CD4 Naive cells. (O) Mean scATAC-seq coverage at *IFNG* loci in CD8 Naive cells. (P) Mean scATAC-seq coverage at *DUSP5* loci in CD8 Memory cells. (Q) Mean scATAC-seq coverage at *PDCD1* loci in NK3 cells

After quality control, a total of 74,102 cells (33,004 YA, 41,098 AA) were used to generate a PBMC chromatin-accessibility map. MEGAs, TCs, NKs, BCs and myeloid cells were identified based on the promoter sum of genes specifically upregulated in each cluster. After separately reclustering each lineage population, we identified 3 distinct subsets in CD4^+^ TCs, 3 distinct subsets in CD8^+^ TCs, 3 distinct subsets in NKs, 3 distinct subsets in BCs, 3 distinct subsets in DCs and 2 distinct subsets in MCs according to gene peaks and transcription factor (TF) activity using chromVAR (Satpathy et al., 2019) (Fig. 4A, 4B, and S8A–D, see Table S 3C for the details). Consistent with the scRNA-seq and CyTOF data, we observed a decrease in naive TCs and an increase in MCs in the elderly (Fig. 4C).

Next, we focused on the differentially expressed transcription factors (DETs) in immune cells in the AA group compared to the YA group. At the TF level, MCs were the most affected by aging based on the numbers of upregulated and downregulated DETs (Fig. 4D and 4E). To identify aging-associated TF events, we performed an integrated comparative analysis of these DETs and found that AP-1 family TFs, including *FOSL2* and *JUNB*, were increased in all immune cells during aging (Fig. 4D and 4E). Upregulation of AP-1 family TFs, including *FOS*, *FOSB*, *FOSL1*, *FOSL2*, *JUN*, *JUNB*, and *JUND*, was also observed in almost all cell subsets during aging (Fig. 4F and 4I). The AP-1 family regulates a wide range of cellular processes, including cell proliferation, death, survival, and differentiation. The effects of the activated AP-1 TFs, associated with the active inflammatory state, are primarily mediated through combinatorial regulation with the NFAT family, both of which are key regulators of TC activation and are enriched in TCs (Fig. 4D) (Shaulian and Karin, 2002). In addition, we visualized the chromosomal accessibility of *FOSL2* loci and *NFATC2* loci and found that the chromosomal accessibility of the *FOSL2* and *NFATC2* gene regions was also increased in aged TCs (Fig. 4J and 4K). *CDKN2B*, an aging hallmark gene, also showed an increase in accessibility with age (Fig. 4L). In parallel, we found 25 common decreased TFs, including nuclear respiratory factor 1 (*NRF1*) and *ELK4*, which are involved in antioxidant stress and negatively regulate cell differentiation and proliferation (Figs. 4E and S8E–I). Consistently, we found that chromatin accessibility also decreased at the loci of *SIRT7* (Fig. 4M), which coordinates with *NRF1* to maintain cellular energy metabolism and proliferation (Mohrin et al., 2015).

In TCs, a series of subset-specific TF changes were observed, such as *GLI2* in naive cells, which has been associated with decreased TC function and impaired immune defenses (Fig. 4F and 4G). Consistently, increased chromatin accessibility was detected in *GLI2* loci (Fig. 4N). Analysis of differentially accessible regions (DARs) demonstrated that the *IFNG, DUSP5*, and *GZMB* loci were highly accessible, which indicated activated CD8^+^ TC states (Figs. 4O, 4P and S8J). In our analysis of NK status, we identified the key TF changes in NK subsets during aging (Fig. 4H), and found that the chromatin accessibility of the inhibitory receptor gene increased, while that of the activating receptor decreased. These changes may weaken the ability to clear virus-infected cells. For example, the *PDCD1* exhibited higher chromatin accessibility in the gene region of the elderly group, which might be part of the reason why older individuals were prone to infection (Fig. 4Q). In our analysis of BCs, we identified aging-related TF changes, such as *TBX21*, *IRF4*, which are consistent with our scRNA-seq results (Fig. 4I). Aging-associated TFs and DARs in MCs demonstrated enrichment in inflammatory-related TFs and gene loci in the AA group, such as NF-κB family (*REL*, *RELA*), *IL1B*, *TNF* and *CXCL8* (Figs. 4I and S8K–M). In summary, aging-related chromosomal accessibility changes are associated with an increase in the inflammatory pathway and an impaired immune response.

### Aging-associated heterogeneous changes in clonality and diversity of TCRs and BCRs

**Figure 5:**
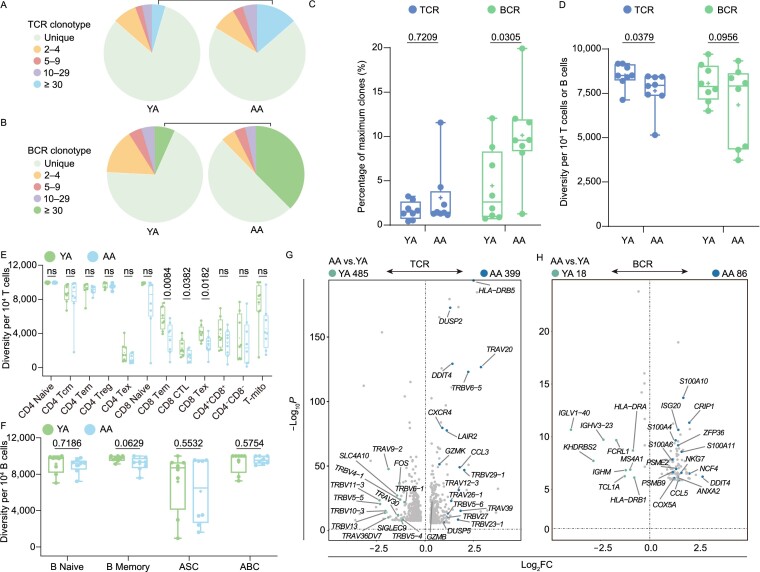

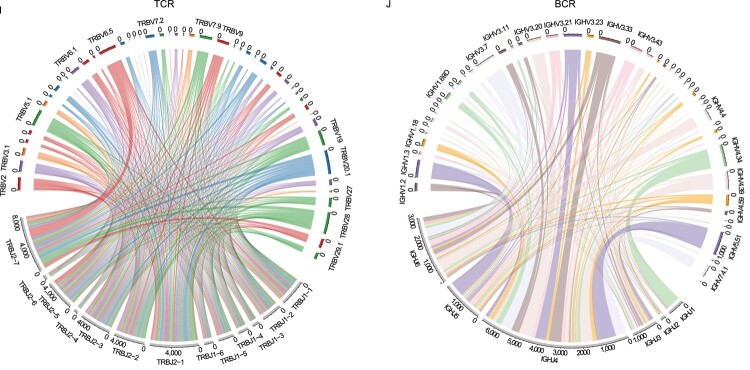
**Abnormal TCR and BCR repertoire during aging**. (A) Pie plots showing TCR clone differences across YA and AA groups. (B) Pie plots showing BCR clone differences across YA and AA groups. (C) Percentage of maximum clones between YA (*n* = 8) and AA groups (*n* = 8). (D) Diversity of TCR and BCR between YA (*n* = 8) and AA groups (*n* = 8). (E) Diversity of TCR in T cell subsets between YA (*n* = 8) and AA groups (*n* = 8). (F) Diversity of BCR in B cell subsets between YA (*n* = 8) and AA groups (*n* = 8). (G) Volcano plot showing DEGs of clonal T cells between the YA and AA groups. *P* values were calculated using a paired, two-sided Wilcoxon test and FDR was corrected using the Benjamini-Hochberg procedure. (H) Volcano plot showing DEGs of clonal B cells between the YA and AA groups. *P* values were calculated using a paired, two-sided Wilcoxon test and FDR was corrected using the Benjamini-Hochberg procedure. (I) Chord diagram showing pairing of V and J segments within the TRB subset from the AA group. Chord widths represent the proportion of sequences with a given V (colored) and J (gray) segment pairing. (J) Chord diagram showing pairing of V and J segments within the IGH subset from the AA group. Chord widths represent the proportion of sequences with a given V (colored) and J (gray) segment pairing

Although the antigen repertoire sensed by immunoglobulin receptors on both TCs (TCRs) and BCs (BCRs) is known to continuously evolve with age (Yuseff et al., 2013), the phenomenon of aging-associated TCR and BCR repertoire constriction has not yet been studied at the single-cell level. Here, we employed scTCR/BCR-seq to assess immune cell clonal expansion in the YA and AA groups. We found that relative to the YA group, the AA group was associated with a substantial decrease in unique clonotypes both in TCRs and BCRs (Fig. 5A and 5B), suggesting that both TCR and BCR clonality increased with age. Moreover, quantification of the most highly expanded (maximum) clone for each research subject showed that the ratios of the maximum clones were higher in the AA group than in the YA group (Fig. 5C). Although an aging-related clonal lymphocyte population may reflect an existing adaptive immunity of the elderly, the overall diversity was decreased in the AA group compared to the YA group (Fig. 5D). Analysis of TCR and BCR distributions across different TC and BC subtypes revealed that loss of repertoire diversity was pronounced in CD8^+^, T-mito and memory BCs of the AA group (Fig. 5E and 5F). To understand how clonally expanded TCs could be affected by aging, we performed DEG analysis of clonal cells between YA and AA groups and revealed increased expression of effector and memory TC signatures, including *GZMB*, *GZMK*, *CXCR4*, *CCL3* and various TCR genes in the aged group (Fig. 5G; Table S12A). In addition, clonal BCs showed aging-associated changes, including increased expression of S100A family genes and decreased levels of naive signature genes such as *IGHM* and *TCL1A* in the aged BCs compared to their young counterparts (Fig. 5H; Table S 12B).

To further explore the aging-associated changes on V(D)J rearrangements in TC and BC, we next examined the frequency of genes (variable region) in the YA and AA groups and found that the frequency of several *TRAVs*, *TRBVs*, *IGHVs*, *IGKVs* and *IGLVs* changed with age (Fig. S9A–E), indicating that TCs and BCs had experienced unique clonal V(D)J rearrangements under the adaptive immune environment of the elderly. When analyzing isotype use in BCR repertoires in the YA and AA groups (Fig. S9F), we found that *IGHA* and *IGHG* were overrepresented in the AA group compared to the YA group, suggesting that aging might induce more frequent isotype switching (Fig. S9G). In addition, the chord diagrams of the V-J arrangement for each group showed that aging resulted in multiple cloning sites, suggesting increased antigen exposure with age (Figs. 5I, 5J, S9H and S9I). The enriched arrangements associated with aging were mainly *TRBV6-5*, *TRBV20-1,* and T*RBV28* in the TRB subset and *IGHV3-33* and *IGHV5-51* in the IGH subset. Taken together, these data show that increased clonality and decreased diversity in aging immune cells, combined with a skewed use of variable region genes, underlie aging-associated abnormalities of TCR and BCR repertoires, elucidating the abnormal immune states and disease spectra during aging.

### Age-related imbalance in cellular composition is associated with poor outcomes in patients with COVID-19

**Figure 6:**
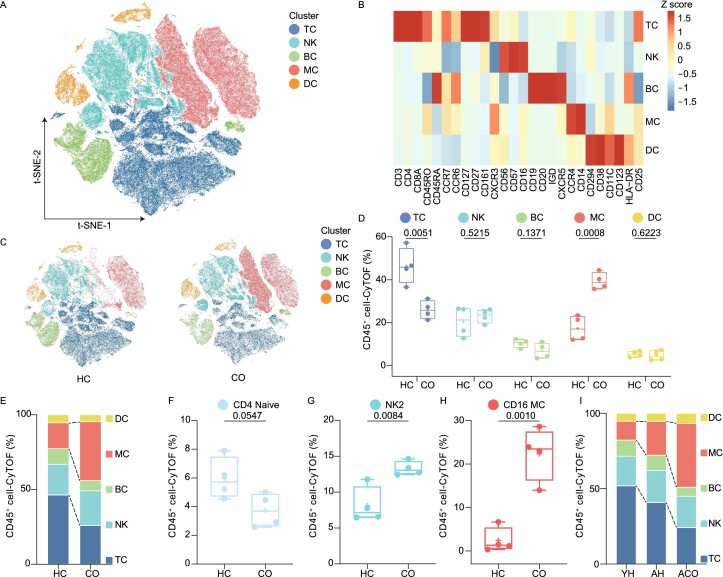

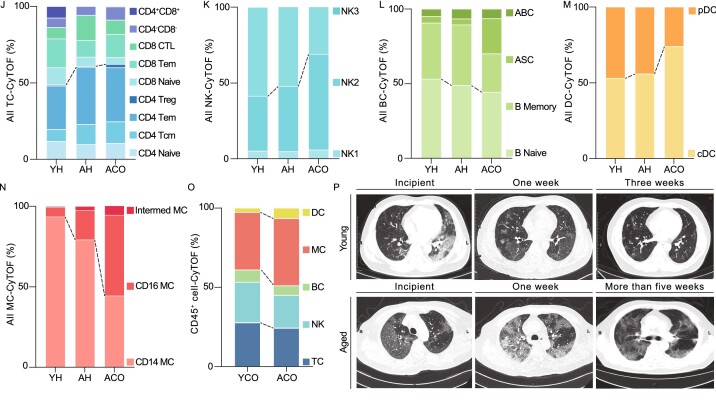
**Poor outcomes upon COVID-19 infection is associated with imbalanced cellular aging**. (A) t-SNE projections of PBMCs derived from mass cytometry data in cohort-2. (B) Heatmap showing mean population expression levels of all markers. (C) t-SNE plots segregated by HC and CO groups. HC includes YH (*n* = 2) and AH (*n* = 2); CO includes YCO (*n* = 2) and ACO (*n* = 2). (D) Percentage of immune cell populations in PBMC between HC (*n* = 4) and CO (*n* = 4) groups. (E) Bar chart of the relative percentage of major immune cell populations derived from mass cytometry data between HC and CO groups. (F) Percentage of CD4 Naive cells in CD45^+^ cells between HC (*n* = 4) and CO (*n* = 4) groups. (G) Percentage of NK2 cells in CD45^+^ cells between HC (*n* = 4) and CO (*n* = 4) groups. (H) Percentage of CD16 monocytes in CD45^+^ cells between HC (*n* = 4) and CO (*n* = 4) groups. (I) Bar chart of the relative percentage of major immune cells derived from mass cytometry data from YH, AH and ACO groups. (J) Bar chart of the relative percentage of T cell subsets derived from mass cytometry data from YH, AH and ACO groups. (K) Bar chart of the relative percentage of NK cell subsets derived from mass cytometry data from YH, AH and ACO groups. (L) Bar chart of the relative percentage of B cell subsets derived from mass cytometry data from YH, AH and ACO groups. (M) Bar chart of the relative percentage of DC subsets derived from mass cytometry data from YH, AH and ACO groups. (N) Bar chart of the relative percentage of monocyte subsets derived from mass cytometry data from YH, AH and ACO groups. (O) Bar chart of the relative percentage of major immune cell populations derived from mass cytometry data between YCO and ACO groups. (P) CT photography showing the different evolution of Lung Ground-Glass Opacity in young and aged patients with COVID-19. CT, computed tomography. *P* values are based on two-tailed Mann-Whitney-Wilcoxon tests between groups

To depict how the immune landscape changes with aging and SARS-CoV-2 infection, we enrolled young (YCO, *n* = 2) and aged (ACO, *n* = 2) patients with incipient COVID-19 (to assess the acute inflammatory state) in cohort-2 and young (YCR, *n* = 2) and aged (ACR, *n* = 2) patients who had recovered from COVID-19 (to assess the recovered state) in cohort-3. In addition, we performed CyTOF analysis of PBMCs from YH, AH, YCO and ACO individuals in cohort-2 (*n* = 2 for each group) (Figs. 6A, S10 and S11). Similar to our CyTOF analysis in cohort-1, we identified 21 clusters: 9 subsets of TCs, 3 subsets of NKs, 4 subsets of BCs, 3 subsets of MCs, and 2 subsets of DCs (Figs. 6B and S11). We first compared the peripheral immune cell composition between COVID-19 patients (at the onset stage, CO) and their age-matched healthy controls (HC). Between the CO and HC groups, we found a similar trend of variation to aging, reflected in a decreased percentage of TCs and increased MC and NK populations (Fig. 6C–E). This trend was also observed at the cell subtype levels, as evidenced by decreased pDC, naive and memory TCs and BCs and increased populations of effector TCs, CD16 MCs, intermediate MCs, ASCs and ABCs (Figs. 6F, 6H, and S12A–L). Importantly, the aging-associated increase in MCs and decrease in TCs were amplified by COVID-19 in aged patients compared with healthy aged controls (Fig. 6I). This trend was also observed at the cell subtype level, as reflected by decreased naive TCs and BCs and increased populations of effector TCs, CD16 MCs, ASCs and ABCs in each immune cell composition and total circulating immune cells (Figs. 6J–N and S12M).

Notably, we found a higher ratio of MCs, especially inflammatory MCs, and a lower percentage of TCs in aged COVID-19 patients than young COVID-19 patients (Figs. 6O and S12N). Notably, comparative subgroup analysis demonstrated that naive BCs and pDCs were decreased in aged patients (Fig. S12O–S). The patients in cohort-2 were diagnosed with severe COVID-19 and presented with similar clinical symptoms and CT findings. Despite these similarities, the recovery and outcomes in the young and aged patients differed substantially. As was evident in high-resolution CT scans, ground-glass opacity in the lungs of young patients gradually dissipated after a period of treatment, but this parameter remained associated with extensive fluid buildup (exudation) and pleural effusion in aged patients (Fig. 6P). Infiltrating MCs can enter the lung and other organs and release abundant levels of inflammatory cytokines and chemokines, exacerbating the infection and leading to fatal outcomes. Aged COVID-19 patients had more MCs and fewer TCs than young patients, thus lowering the threshold of developing hyperinflammatory states that may trigger cytokine storms and lymphopenia.

### Aging increases the expression of susceptibility genes for COVID-19, and COVID-19 enhances upregulation of aging-induced inflammatory genes

**Figure 7:**
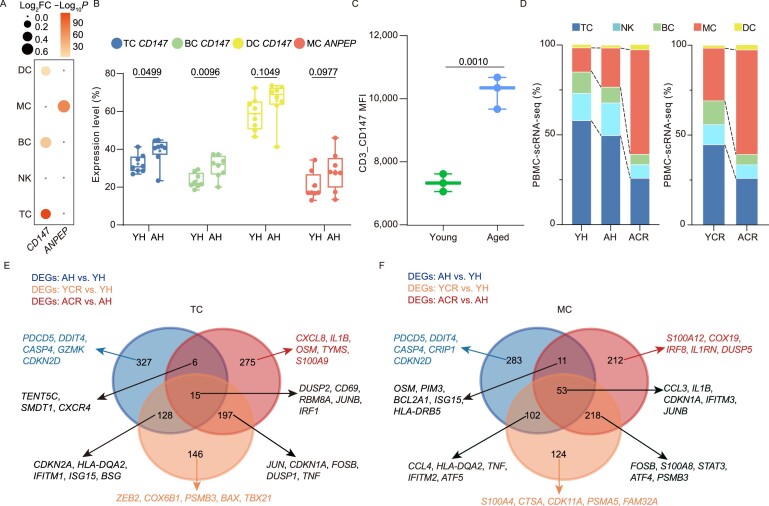

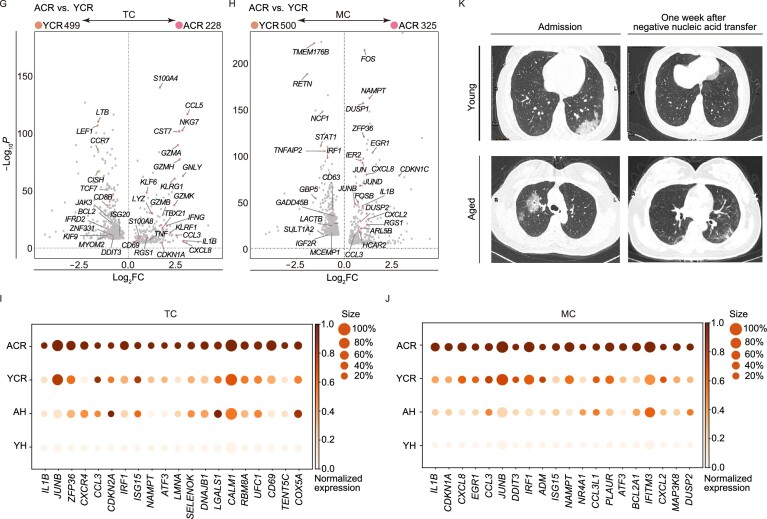
**Aging and SARS-CoV-2 infection are characterized by similar hyper-inflammatory states**. (A) Dot plot showing increased *BSG* and *ANPEP* expression in major immune cell populations in the AH group compared to YH group. *P* values are based on two-tailed Mann-Whitney-Wilcoxon tests between groups. (B) Expression levels of *BSG* and *ANPEP* in specific cell types in YH and AH groups. (C) Recapitulative graph of the MFI of CD147 expression in CD3^+^ T cells. MFI, mean fluorescence intensity. (D) Bar charts of the relative percentage of major immune cell populations derived from scRNA-seq data in YH, AH and ACR group (left), YCR and ACR group (right). (E) Venn diagram showing the integrated comparative analysis of upregulated DEGs in T cells between YH and AH group, YCR and YH group, ACR and AH group. The count shows the number of DEGs. (F) Venn diagram showing the integrated comparative analysis of upregulated DEGs in monocytes between AH and YH groups, YCR and YH groups, ACR and AH groups. The count shows the number of DEGs. (G) Volcano plot showing DEGs in T cells between YCR and ACR groups. *P* values were calculated using a paired, two-sided Wilcoxon test and FDR was corrected using the Benjamini-Hochberg procedure. (H) Volcano plot showing DEGs in monocytes between YCR and ACR groups. *P* values were calculated using a paired, two-sided Wilcoxon test and FDR was corrected using the Benjamini-Hochberg procedure. (I) Dot plot showing expression levels of the top 20 aging-induced and disease-associated genes in T cells per group in cohort-3. (J) Dot plot showing expression levels of the top 20 aging-induced and disease-associated genes in monocytes per group in cohort-3. (K) CT photography showing the different manifestation of evolution of Lung Ground-Glass Opacity in young and aged patients with COVID-19

To determine how an increased MCs population and decreased TCs population at the onset of SARS-CoV-2 infection contribute to faster disease progression in the elderly at the cellular and molecular levels, we used scRNA-seq to investigate the association between aging and COVID-19. Specifically, we analyzed DEGs to explore whether differentially expressed SARS-CoV-2-related genes in aged patients could explain the impact that aging had on the susceptibility and recovery of COVID-19 patients in cohort-3 (Figs. S13A–C and S14A). ACE2 is not expressed by any blood immune cells, and recent studies have reported that CD147 (encoded by *BSG*), CD26 and ANPEP might be alternative cellular entry receptors for SARS-CoV-2, especially CD147, in TCs (Han et al., 2020; Qi et al., 2020; Ulrich and Pillat, 2020). Anti-CD147 antibody has been tested to treat COVID-19 patients with promising effects (Bian et al., 2020). We found that *BSG* expression in the AH group was increased in TCs, BCs and DCs, while *ANPEP* was only upregulated in MCs (Fig. 7A). Moreover, we found that aging increased the frequency of immune cells that expressed *BSG* and *ANPEP* (Fig. 7B). This result was validated using flow cytometry analysis, which showed increased CD147 expression in CD3^+^ TCs in the aged people compared with the young group (*P* = 0.0010, Fig. 7C). We further observed higher expression of the CD147-related genes *NFATC1*, *ITGB1*, and *PPIB* in CD4 Naive of the AH group (Fig. S14B–D). In addition, CD26 (encoded by *DPP4*), another potential SARS-CoV-2 receptor (Radzikowska et al., 2020), was only upregulated in CD4 Naive of the AH group (Fig. S14E). Altered expression of these molecules in circulating immune cells, especially in CD4 Naive, with age might contribute to increased susceptibility and severity of COVID-19 in the elderly.

Upregulation of SARS-CoV-2-related genes in aged individuals indicates that aging increases susceptibility to this infection. Our data demonstrated that the inflammatory response was sustained in the blood environment of COVID-19 patients recovering from SARS-CoV-2 infection (Wen et al., 2020). In the recovery stage, the aging-associated increase in MCs and decrease in TCs were amplified by COVID-19 in aged covered patients compared with healthy aged controls. Importantly, the ACR group still had more MCs and fewer TCs than the YCR group (Figs. 7D and S14F). To compare the effects of age on disease recovery, we next analyzed the upregulated DEGs between the YCR and YH groups and between the ACR and AH groups, along with combined analysis of the upregulated aging-related DEGs (Table S 13-14). We identified aging-induced and disease-associated genes in TCs, including *CD69*, *JUNB*, *CDKN2A*, and IFN-related genes, including *IRF1* and *ISG15* (Fig. 7E). In MCs, we identified several aging-induced genes involved in disease development, such as *TNF*, *IL1B*, *JUNB*, *DUSP2*, *OSM*, the *CDKN* family, IFN-related genes, and chemokine family members (Fig. 7F). Analysis of the DEGs between TCs of the YCR and ACR groups demonstrated that several granzyme genes and inflammatory genes were increased in aged recovered patients (Fig. 7G). Moreover, we found that MCs in the ACR group had increased expression of inflammatory genes such as *FOS*, *DUSP1*, *IL1B*, and *JUN* and chemokines including *CXCL8* and *CCL3* compared to those of the YCR group (Fig. 7H). We finally compared the expression of the top 20 specific aging-induced and disease-associated genes among the 4 groups in MCs and TCs, respectively (Fig. 7I and 7J). The results showed that COVID-19 amplifies aging-induced upregulation of inflammatory genes and senescence hallmark genes (*CDKN* family) (López-Otín et al., 2013). As expected, although the initial clinical manifestations and diagnosis were similar, lung ground-glass opacity in young patients had been dissipated and absorbed completely, but in aged patients, it was not absorbed completely at one week after a negative nucleic acid test (Fig. 7K). These findings indicate that aged people have a slower recovery from COVID-19 than young people.

We next predicted cell-to-cell interactions that might contribute to the distinct functional status of circulating TCs and BCs of the YCR and ACR patients (Fig. S15). In ACR patients, we discovered that TCs expressed high levels of *IFNG*, the ligands for *IFNGR1*, which was expressed on MCs (Fig. S15A). Other TC-MC interactions involved the inflammatory response, cell-cell signaling and cell adhesion. Notably, TCs might activate MCs through the expression of *CCL5* ligands that bind to *CCR1* and contribute to inflammatory activation. Interestingly, TCs in the ACR patients expressed high levels of *IL-4*, which was predicted to bind *IL-4R* and *IL-2R* in TC-MC interactions and was reported to enhance viral infection (Rogers et al., 2019). In the ACR group, BCs expressed increased levels of genes encoding ligands of *IL1R* and *TNFRSF1B* (Fig. S15B). The expression of these molecules in MCs suggests that BCs may contribute to the activation of IL1B and TNF signaling in circulating MCs. Compared with the ACR group, the YCR group was characterized by the presence of signals that negatively regulate the inflammatory response molecules *IL10*-*IL10RA* in TC or BC interactions with MCs. Downregulation of negative regulatory signals may also contribute to the slow dissipation of inflammation in the elderly. Overall, enhanced inflammatory signals and impaired regulatory signals between TCs and MCs, or, BCs and MCs, slow recovery in elderly patients.

## DISCUSSION

Here, we present a comprehensive and integrated single-cell landscape of human circulating immune cell aging and single-cell analysis of immune cells in young and aged COVID-19 patients at the transcriptomic and protein level. The primary discoveries in the current study are as follows: 1) aging reprograms the human immune cell landscape toward polarized and inflammatory states; 2) aging increases the expression of SARS-CoV-2 susceptibility genes, especially in TCs; 3) an increase in immune cell polarization and circulatory inflammation during aging can be amplified by virus infection in COVID-19; 4) age-associated dendritic cells have increased IFN-stimulated gene expression and a decreased antigen-presenting ability; 5) single-cell TCR and BCR analysis shows that aging is associated with decreased diversity and increased clonality of effector, cytotoxic and exhausted CD8^+^ TC subsets and ABC subset; 6) single-cell chromosomal accessibility profiles of immune cells shows that the AP-1 family TFs are the most affected by ageing across all cell types and subtypes and are further upregulated in COVID-19.

Numerous studies have reported important observations about the composition and functional alterations of immune cells in animal aging models. However, animal models fail to recapitulate the human immune environment adequately. What we know about human immune cells is primarily based on flow cytometric analysis, relying on previously described markers for pooled cell populations. These analytical methods are too biased to reveal information on selected and not all cells or cell populations. Single-cell technologies open new avenues in many research fields but are particularly important for analyzing human cells in aging and diseases in an unbiased and global fashion (He et al., 2020; Wang et al., 2020; Zhang et al., 2020). Using scRNA-seq, recent studies have reported transcriptomic and functional changes in immune cells during aging in mouse cells and tissues such as the central nervous system (Mrdjen et al., 2018), macrophages in brain (Martinez-Jimenez et al., 2017; Van Hove et al., 2019), TCs in spleen (Dulken et al., 2019), and hematopoietic stem cells in bone marrow (Leins et al., 2018). Recently, our group revealed how aging affects the immune system in rats (Ma et al., 2020). For humans, mass cytometry analysis showed that aging increased epigenetic variations in circulatory immune cells (Cheung et al., 2018). However, a comprehensive atlas of immune cell aging has not yet been constructed. Here, we depicted such an atlas from PBMCs harvested from healthy young and old research subjects and young and old patients with COVID-19. First, scRNA-seq and CyTOF reveal that aging causes cell compositional changes at the cell type and subtype levels. Second, our study provides the first high-quality analysis of TCR and BCR repertoires in young and aged adults at a single-cell resolution. Third, our study provides the first chromosomal accessibility profiles of major immune cells in young and aged healthy research subjects at the single-cell level. Combined with several novel single-cell methodologies, this study represents a state-of-the-art unbiased and systematic analysis of human immune cell aging.

Mechanistically, we observed age-associated alterations in immune cell type and subtype composition, gene expression, transcriptional regulation, chromosomal accessibility, TCR and BCR repertoires, and cell-cell communication across multiple cell types and subtypes. Our data suggest that increased numbers of MCs may contribute to cytokine storms in coronavirus infection, as indicated by increased numbers of MCs during aging and further increases in COVID-19, whereas TCs that are critical for virus clearance (Hickman et al., 2015; Herzig et al., 2019) were decreased during aging and further reduced in COVID-19. Through the analysis of cell subtype composition, we found that naive subsets were profoundly decreased with age, likely weakening the responsive capacity of TCs during viral infection. In addition, the polarization from naive to effector cells was further enhanced by SARS-CoV-2 infection in COVID-19. Inflammatory genes such as *IL1B*, *TNF*, and *CXCL8* were also increased during aging and were further upregulated in COVID-19. Notably, aging promoted the expression of coronavirus receptor-related genes, such as *BSG* (encoding CD147), *DPP4* (encoding CD26), *ITGB1*, *NFATC1*, *PPIB* and *ANPEP,* in immune cells. Collectively, these findings reveal that aging reprograms the landscape of human immune cells toward polarized and inflammatory states and thus increases the susceptibility of COVID-19 in the elderly. In turn, COVID-19 causes more “aging” of polarization and inflammatory states in immune cells. This reinforcing feedback loop may underlie the immune system collapse in aged people.

Due to technical limitations, high-dimensional molecular profiles in aging for rare cells such as DCs are lacking. Here, we overcame this challenge with a novel single-cell method. Aging increased the percentage of cDC2 cells and decreased the percentage of pDCs that engage antiviral activities by priming CD8^+^ TCs. By comparison, aging decreased the expression of *CLEC12A*, *TXNIP*, *AHR* and *MALAT1* and increased the expression of *HLA-DQA2* and IFN-stimulated genes. *CLEC12A* (Hutten et al., 2016) and *TXNIP* (Son et al., 2008) are critical for the antigen-presentation function of DCs, whereas *MALAT1* and *AHR* are critical for tolerogenic DC differentiation (Takenaka and Quintana, 2017; Wu et al., 2018), and their dysregulation hampers DC function. Interestingly, *HLA-DQA2* and IFN-stimulated genes were distinctly expressed in the cDC2 subset during aging. Moreover, our functional analysis of DEGs indicates that the aging of DCs was associated with a decrease in the antigen-presenting ability and an increase in activation of inflammatory signaling pathways, such as the response to hypoxia and IFN signaling. These findings highlight how aging affects DCs composition and function.

**Figure 8:**
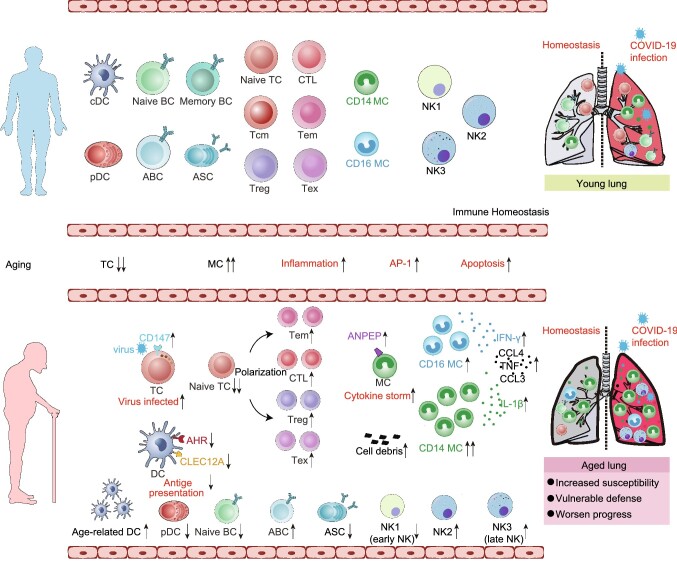
**Aging reprograms human immune cell landscape, and increases the susceptibility and vulnerability of COVID-19**. Schematic illustrating the key innate and adaptive immune functional alterations identified in PBMCs influenced by aging and COVID-19. Young healthy individuals maintain homeostasis in immune system which could timely eliminate pathogen. Aging leads to the increase of monocytes (MCs) and the decrease of T cells (TCs) in the immune system. Aging promotes the polarization of TCs from naive and memory to effector, exhausted and regulatory subtypes and increases the numbers of late natural killer (NK) cells, age-associated B cells, inflammatory MCs, and dysfunctional dendritic cells (DCs). Moreover, aging induces increased expression of genes related to SARS-CoV-2 susceptibility, suggesting increased susceptibility in the elderly. Importantly, aging induces DCs to lose the antigen-presenting ability, and turn to an inflammatory state. Together, a dysregulated immune system and increased expression of genes associated with SARS-CoV-2 susceptibility may at least partially account for COVID-19 vulnerability in the elderly

In this study, we provide a comprehensive atlas of human circulating immune cell aging. Furthermore, we reveal novel aging-related genes and adaptive immune dysregulation, thus defining the potential contributions of aging-related immune cell disorganization to the high severity rate of aged COVID-19 patients (Fig. 8). We believe that these findings will serve as a foundation from which to explore unknown facets of aging etiology and a reference for the broad scientific community interested in immunology and aging.

## Materials and Methods

### Human subjects

The study was approved by the Ethics Committee of Zhongshan Ophthalmic Center, China and the Ethics Committee of Wuhan Hankou Hospital, China. A written informed consent was routinely obtained from all individuals participating in the study and all relevant ethical regulations regarding human research participants were followed. Healthy non-frail individuals were recruited in the Zhongshan Ophthalmic Center, and divided by age into two groups in cohort-1: young adults (YA) and aged adults (AA). The YA group ranged from ages 20 to 45 years old and the AA group ranged from ages 60 to 80 years old. COVID-19 patients diagnosed by real-time fluorescent quantitative reverse transcription polymerase chain reaction (RT-qPCR) and CT images were enrolled in the Wuhan Hankou Hospital, China. Based on their clinical history, patients were divided into incipient and recovered groups in cohort-2 and cohort-3 respectively, and the incipient hospitalized patients were further divided by age into young COVID-19 patient onset (YCO) and aged COVID-19 patient onset (ACO). Enrolled patients that tested negative with nucleic acid transfer in 7–14 days were further divided into young COVID-19 patient recovered (YCR) and aged COVID-19 patient recovered (ACR). Individuals with comorbid conditions including cancer, immunocompromising disorders, hypertension, diabetes and steroid usage were excluded. No significant gender differences were detected between YA group and AA group in cohort-1 (Table S1C–E), between YH, AH, YCO and ACO group in cohort-2 (Table S1F), between YH, AH, YCR and ACR group in cohort-3 (Table S1G).

### Antibodies and reagents

Antibodies against the following markers in flow cytometric analysis were purchased from Biolegend, BD biosciences and Abcam: CD3 (clone SK7) BV785 (Cat. 344842), CD19 (clone HB19) APC (Cat. 302212), CD88 (clone S5/1) PE/Cy7 (Cat. 344307), CD89 (clone A59) PE/Cy7 (Cat. 354107), HLA-DR (clone L243) FITC (Cat. 307604), CD11c (clone 3.9) BV421 (Cat. 301627), FcεRIa (clone AER-37) PercP/Cy5.5 (Cat. 334622), CD1c (clone L161) BV650 (Cat. 331541), CD371 (CLEC12A) (clone 50C1) PE (Cat. 353603) were purchased from Biolegend, CD147 (clone HIM6) PE (Cat. 562552) was purchased from BD biosciences. Fetal bovine serum (FBS) (Cat. 10270-106), penicillin/streptomycin (Cat. 15140-122), and Trypsin-EDTA (0.25%) (Cat. 25200-072) were purchased from GIBCO. RT-qPCR kit (Cat. 25200-072) was purchased from TaKaRa.

### Detection of SARS-Cov-2 with RT-qPCR

Samples used for RT-qPCR were blood, upper respiratory tract sputum and throat swab obtained from patients at specified time-points during hospitalization. The patient samples were collected, processed and analyzed following the guideline stipulated by the WHO. To extract viral RNA, the specimens were treated with the QIAamp RNA Viral Kit (Qiagen, Heiden, Germany) following the manufacturer’s guidelines. The presence of SARS-CoV-2 infection was confirmed with a China CDC recommended RT-qPCR kit (TaKaRa, Dalian, China). qPCR was performed as previously described (Zhang et al., 2019; Bi et al., 2020; Li et al., 2020).

### Isolation of PBMCs for mass cytometry, scRNA-seq and scATAC-seq

For pipeline analysis, venous blood samples were derived from each healthy donor or patient using Ficoll-Hypaque density solution, heparinized and then processed by standard density gradient centrifugation methods to isolate PBMCs. The viability and quantity of PBMCs in single-cell suspensions were determined using Trypan Blue. For each sample, the cell viability exceeded 90%. For each sample with more than 1 × 10^7^ viable cells, a fraction of PBMCs was extracted for scRNA-seq analysis, a fraction of PBMCs was allocated for scATAC-seq and mass cytometry.

### Flow cytometric analysis

PBMCs suspended in phosphate buffered saline (PBS) were cultured with Live/Dead yellow dye (Invitrogen) at 4 °C for 30 min and then washed once with 1 mL of PBS containing 1% FBS (GIBCO, Grand Island, NY, USA). Subsequently, cells were treated with antibodies for 30 min at 4 °C. These antibodies included: CD3-BV785 (clone SK7, Biolegend), CD19-APC (clone HB19, Biolegend), CD88-PE/Cy7 (clone S5/1, Biolegend), CD89-PE/Cy7 (clone A59, Biolegend), HLA-DR-FITC (clone L243, Biolegend), CD11c-BV421 (clone 3.9, Biolegend), FcεRIa- PercP/Cy5.5 (clone AER-37, Biolegend), CD1c-BV650 (clone L161, Biolegend), CD147-PE (clone HIM6, BD biosciences), CD371 (CLEC12A)-PE (clone 50C1, Biolegend). Analysis of PBMCs with flow cytometry was conducted with BD Fortessa (BD Biosciences) and the results were evaluated with FlowJo (version 10.0.7, Tree Star, Ashland, OR, USA).

### Mass cytometry live cell barcoding and surface staining

We made use of a live cell barcoding approach to minimize inter-sample staining variability, sample handling time and antibody consumption. After incubating with anti-human CD45 loaded with different isotopes (89Y, 162Dy, 165Ho, 169Tm, 175Lu), all the samples were then pooled for surface staining. The Maxpar Direct Immune Profiling Assay (Fluidigm) was used for cell surface staining and the monoclonal anti-human antibodies in the assay kit are listed as Table S2.

Barcoded and combined samples were washed and stained with viability dyes cisplatin-195pt (0.5 μmolL) (Fluidigm, 201064) and vortexed to mix thoroughly for 2 min at room temperature for cell viability, terminated with Maxpar Cell Staining buffer at room temperature (400 *rcf.*), washed, fixed with 1.6% paraformaldehyde (PFA; Electron Microscopy Sciences) in PBS for 10 min at room temperature on a rotary shaker (500 rpm). The fixed cells were resuspended in pre-cooling Maxpar Cell Staining to slow fix reaction. Fixed samples were washed twice with PBS/bovine serum albumin and once with double-distilled water before resuspended in 400 μL of surface-antibody mixture. Surface staining was performed for 30 min at 37 °C on a rotating shaker (500 rpm). The samples then stored in freshly diluted 2% formaldehyde (Electron Microscopy Sciences) in PBS containing 0.125 nmol/L iridium 191/193 intercalator (Fluidigm, 201192) at 4 °C overnight.

### scRNA-seq data alignment, processing and sample aggregation

The Chromium Single Cell 5′ Library (the 10x Genomics chromium platform Illumina NovaSeq6000), Gel Bead and Multiplex Kit, and Chip Kit (10x Genomics) were used to convert single-cell suspension samples to barcoded scRNA-seq libraries. Single-cell RNA libraries were prepared using the Chromium Single Cell 5′ v2 Reagent (10x Genomics, 120237) kit as per the manufacturer’s protocols. The quality of the libraries was checked using the FastQC software. Initial processing of the sequenced data was performed using CellRanger software (https://support.10xgenomics.com, version 3.1.0).

The command Cell Ranger count in CellRanger Software Suite (10x Genomics) was used to demultiplex and barcode the sequences derived from the 10x Genomics single-cell RNA-seq platform. The data was filtered, normalized, dimensionality was reduced, clustered, and differential gene expression analysis were performed after calculation of the single-cell expression matrix by CellRanger using Python (version 3.7.7) Scanpy (https://scanpy.readthedocs.io/en/stable/index.html, version 1.4.6). Data collection and the subsequent analyses were performed in an unsupervised manner, but not blinded to the conditions of the experiments. For quality control, the filtered cell population was mainly those cells that express *HBB*, *HBA1*, and several light and heavy chain transcripts, which identified as the RBC-contaminated cell population. Likewise, several clusters expressing genes has no significance (*P* ≥ 0.1, calculate by 10x genomics Loupe Cell Browser with it default algorithm. *P* values are adjusted using the Benjamini-Hochberg correction for multiple tests) were removed. A total of 16 libraries were sequenced, and 166,609 cells (YA 77,652 cells, AA 88,957 cells) were analyzed after quality control in cohort-1. For cohort-3, 22 libraries and 205,434 cells (YH 79,039 cells, AH 88,750 cells, YCR 19,533 cells, ACR 18,112 cells) were remained for the subsequent analysis. The genes used in principal component analysis (PCA) analysis have eliminated mitochondria (*MT*), and ribosomes (*RPL* and *RPS*) genes with 50 principal components, and then aligned together, followed by t-distributed stochastic neighbor embedding (t-SNE) are both used after the results of the aligned. And using the run_harmony function (in pyharmony package, version 1.0.7) and combat function (in Scanpy) methods to deal with batch effect issues if batch effect existing in dataset. Genes not detected in any cell were removed from subsequent analysis.

### Dimensionality reduction and clustering analysis of scRNA-seq datasets

To analyze the scRNA-seq data, we log normalized data (1 + counts per 10,000) with the ‘‘sc.pp.normalize_total’’ function before clustering, reduction and performing 2-dimensional t-SNE algorithm clustering with the first 50 principal components. This was done following PCA on top 5,000 most variable genes by using “sc.pp.highly_variable_genes” function in Scanpy with the default parameters. Dimensionality method and identification of significant clusters and was performed using Leiden clustering algorithm which uses a shared nearest neighbour modularity optimization-based clustering algorithm. Marker genes for each significant cluster were found using the function sc.tl.rank_genes_groups with default parameters.

### Differential expression analysis

Differential expression analysis for each cell type between different groups (YA and AA in cohort-1 and YH, AH, YCR and ACR in cohort-3) was performed using the *t*-test as implemented in the ‘‘sc.tl.rank_genes_groups’’ function of the Scanpy package. For each cluster, differentially-expressed genes (DEGs) were performed using the *t*-test and generated relative to all of the other cells. Before executing the differential expression analysis, we filtered out the cell types that were missing or had fewer than three cells in the comparison groups. An aging-associated and disease-related DEG dataset was established (adjusted *P* value < 0.05, |Log_2_FC| > 0.25) after identification of DEGs between AA and YA groups in cohort-1, AH and YH groups in cohort-3, ACR and AH groups in cohort-3, YCR and YH groups in cohort-3. The ‘‘upregulated DEGs during aging’’ were defined as the DEGs that increased in AA group and decreased in YA group. The ‘‘downregulated DEGs in aging’’ were defined as the DEGs that decreased in AA group and increased in YA group.

### Gene functional annotation

The Metascape webtool (www.metascape.org) (Zhou et al., 2019) that allow visualization of functional patterns of gene clusters and statistical analysis was used to conduct DEGs gene ontology, pathway enrichment analyses. Among the top 30 enriched GO terms or pathways across various types of cells and tissues, 10 GO terms or pathways which were associated with aging were visualized. Gene expression profile cluster plots and heatmaps were established using the pheatmap R package (https://cran.r-project.org/web/packages/pheatmap/index.html, version 1.0.12).

### Aging score analysis

To assess the impact of aging in circulating immune cells, we selected the top 20 genes out of 60 common upregulated genes in all immune cells. Aging scores were estimated for all cells as the average of the scaled (Z-normalized) expression of the genes in the list. The score of aging for all immune cell types can be used to predict the effect of aging on single cells and cell subtype levels. Calculation of the scores was done as follows: the score of the aging gene set in the given cell-subset (named as X) was computed as the sum of all UMI for all the aging genes expressed in X cell, divided by the sum of all UMI expressed by X cell (Pont et al., 2019).

### Sequencing and analysis of TCR and BCR V(D)J

PCR amplification was done to enrich the full-length TCR/BCR V(D)J segments for the amplified cDNA from 5′ libraries with a Chromium Single-Cell V(D)J Enrichment kit (10 Genomics). The TCR/BCR sequences of each T/B cell were clustered using the CellRanger vdj pipeline (version 3.1.0, allowing identification of CDR3 sequence and the rearranged TCR/BCR gene. Analysis was performed using Loupe V(D)J Browser version 2.0.1 (https://support.10xgenomics.com, 10x Genomics). In summary, barcode information a containing clonotype frequency and TCR/BCR diversity metric were obtained. We projected T /B cells with dominant TCR/BCR clonotypes on a t-SNE plot using barcode information (Wen et al., 2020).

### Determination of cell-cell interaction

We employed the expression of immune-related ligands and receptors to assess the cell-cell interactions (Ma et al., 2020). The possible ligand-receptor interactions between one set of receptor-expressing cells and then next ligand-expressing cells were determined as the average of the product of receptor and ligand expression (respectively from set one and two) across all single-cell pairs:

$$I=\sum_{i}^{n}I_{i}\times \sum_{j}^{m}r_{j}\left(\frac{1}{m*n}\right)$$

where *I* refers to the interaction score between receptor expressing cells in set one and ligand-expressing cells in set two, *Ii* stands for the ligand expression of cell *i* in cell set one, *rj* represents the receptor expression of cell j in cell set two, *n* stands for the number of cells in set one and m denotes the number of cells in set two.

In the gene list, there were 168 pairs of well-annotated ligands and receptors, among which were co-stimulators, chemokines and cytokines. The possible interactions between two cell types were orchestrated by receptor-ligand pairs by the product of the average expression levels of the ligand in one cell type and the respective receptor in the other cell type.

### Mass cytometry processing and quality control

CyTOF data were acquired with a CyTOF2 system using a SuperSampler fluidics system (Victorian Airships) at an event rate of < 400 events per second and normalized with Helios normalizer software (Fluidigm version 6.7.1016). Acquisitions from different days (three independent acquisitions were performed) were normalized using five-element beads (Fluidigm). Barcoded samples were deconvoluted and cross-sample doublets were filtered using cytobank application. CyTOF data was pre-processed with Cytobank (https://mtsinai.cytobank.org; Cytobank, 7.0) to sequentially remove calibration beads, dead cells, debris and barcodes for CD45^+^ PBMCs based on event length, DNA (191Ir and 193Ir) and live cell (195Pt) channels and then export the FCS files. We analyzed 200,000 PBMCs in cohort-1 and 160,000 PBMC in cohort-2, with an average of 20,000 cells per sample.

### Mass cytometry visualizing and clustering

We created mass cytometry datasets for analysis by concatenating cells from all individuals for each cell type. In this way, we created downsampled datasets of 95,316 TCs, 35,254 NKs, 22,042 BCs, 39,144 MCs and 8,244 DCs in cohort-1 and 57,910 TCs, 34,857 NKs, 13,812 BCs, 45,431 MCs and 7,990 DCs in cohort-2 for analysis. We used FlowCore (65 *flowCore: Basic structures for flow cytometry data*.) to read and process FCS files for further analysis. For sample with more than 20,000 cells, we randomly selected 20,000 cells to ensure that samples were equally represented. At last, we run the t-SNE dimensionality reduction algorithm on a combined data sample using the Seurat package based on harmony embedding (https://github.com/immunogenomics/harmony, version 1.0.0).

### Batch correction of mass cytometry data

PBMC mass cytometry data from 10 subjects of cohort-1 or 8 subjects of cohort-2 was combined and batch normalized using harmony respectively. First, mass cytometry data from each cohort all subjects was combined into a single dataset. Second, harmony batch correction was performed using one of the samples. Third, mass cytometry data were lognormalized in the Seurat’s NormalizeData function across the aggregated dataset.

### Single-cell assay for transposase-accessible chromatin sequencing (scATAC-seq)

scATAC-seq targeting 4,000 cells per sample was performed using Chromium Single Cell ATAC Library and Gel Bead kit (10x Genomics, 1000110). Each sample library was uniquely barcoded and quantified by RT-qPCR. Libraries were then pooled and loaded on an Illumina Novaseq 6000 sequencer (3.5 pmol/L loading concentration, 50 + 8 + 16 + 49 bp read configuration) and sequenced to either 90% saturation or 30,000 unique reads per cell on average. All protocols to generate scATAC-seq data on the 10x Chromium platform, including sample preparation, library preparation and instrument and sequencing settings, are available here: https://support.10xgenomics.com/single-cell-atac.

### scATAC-seq data processing

#### scATAC-seq processing

scATAC-seq reads were aligned to the GRCh38 (hg38) reference genome and quantified using CellRanger-ATAC count (10x Genomics, v.1.0.0).

#### scATAC-seq quality control

To ensure that each cell was both adequately sequenced and had a high signal-to-background ratio, we filtered cells with less than 1,000 unique fragments and enrichment at TSSs < 8. To calculate TSS enrichment > 2, genome-wide Tn5-corrected insertions were aggregated ± 2,000 bp relative (TSS-strand-corrected) to each unique TSS. This profile was normalized to the mean accessibility ± 1,900–2,000 bp from the TSS, smoothed every 51 bp and the maximum smoothed value was reported as TSS enrichment in R. To construct a counts matrix for each cell by each feature (peaks), we read each fragment.tsv.gz fill into a GenomicRanges object. For each Tn5 insertion, which can be thought of as the “start” and “end” of the ATAC fragments, we used findOverlaps to find all overlaps with the feature by insertions. Then we added a column with the unique id (integer) cell barcode to the overlaps object and fed this into a sparseMatrix in R. To calculate the fraction of reads/insertions in peaks, we used the colSums of the sparseMatrix and divided it by the number of insertions for each cell id barcode using table in R.

#### scATAC-seq visualization in genomic regions

To visualize scATAC-seq data, we read the fragments into a GenomicRanges object in R. We then computed sliding windows across each region we wanted to visualize for every 100 bp “slidingWindows (region, 100, 100)”. We computed a counts matrix for Tn5-corrected insertions as described above and then binarized this matrix. We then returned all non-zero indices (binarization) from the matrix (cell × 100-bp intervals) and plotted them in ggplot2 in R with “geom_tile”. For visualizing aggregate scATAC-seq data, the binarized matrix above was summed and normalized. Scale factors were computed by taking the binarized sum in the global peak set and normalizing to 10,000,000. Tracks were then plotted in Loupe Cell Browser, an interactive visualization software that shows scATAC-seq peak profiles for scATAC-seq cell clusters, similar to the analysis done in this manuscript and described at https://support.10xgenomics.com/single-cellatac/software/visualization/latest/what-is-loupe-cell-browser.

#### chromVAR

We measured global TF activity using chromVAR15. We used the cell-by-peaks and the Catalog of Inferred Sequence Binding Preferences (CIS-BP) motif (from chromVAR motifs “human_pwms_v1”) matches within these peaks from motifmatchr. We then computed the GC-bias-corrected deviation scores using the chromVAR “deviationScores” function.

### Statistical analysis

The GraphPad Prism Software (version 8.0.2) was employed for data analysis and presentation. All results are presented as means ± SEM. Groups were compared with two-tailed Mann-Whitney-Wilcoxon tests and FDR was corrected using the Benjamini-Hochberg procedure. *P* value was derived by a hypergeometric test in representative GO terms and pathways.

## Electronic supplementary material

The online version of this article (https://doi.org/10.1007/s13238-020-00762-2) contains supplementary material, which is available to authorized users.

## Supplementary Material

13238_2020_762_MOESM1_ESM Supplementary Material

13238_2020_762_MOESM2_ESM

## Data Availability

The single-cell sequencing data is deposited in the Genome Sequence Archive in BIG Data Center, Beijing Institute of Genomics (BIG, https://bigd.big.ac.cn/gsa-human/), Chinese Academy of Sciences, with Project Accession No. PRJCA002865 and GSA Accession No. HSA000203. In cohort-3 scRNA seq, YCR and ACR data were obtained from the Genome Sequence Archive in BIG Data Center, Beijing Institute of Genomics (BIG, http://gsa.big.ac.cn), Chinese Academy of Sciences, with Project Accession No. PRJCA002413 and GSA Accession No. CRA002497.

## Abbreviations

AA, aged healthy adults; ABC, age-associated B cell; ACO, aged COVID-19 onset patients; ACR, aged recovered COVID-19 patients; AH, aged healthy; ASC, antibody-secreting cell; BCs, B cells; CD4 Naive, naive CD4^+^ cells; CD4 Tcm, central memory CD4^+^ T cells; CD4 Tem, effector memory CD4^+^ T cells; CD4 Tex, exhausted CD4^+^ T cells; CD4 Treg, CD4^+^ regulatory T cells; CD8 CTL, cytotoxic CD8^+^ cells; CD8 Naive, naive CD8^+^ T cells; CD8 Tem, effector memory CD8^+^ T cells; CD8 Tex, exhausted CD8^+^ T cells; CyTOF, mass cytometry; DARs, differentially accessible regions; DCs, dendritic cells; DEGs, differentially expressed genes; DETs, differentially expressed transcription factors; GO, Gene Ontology; IFN-γ, interferon-gamma; Intermed, intermediate monocytes; MCs, monocytes; MEGAs, megakaryocytes; NK, natural killer; NK1, *CD16*^−^*CD56*^bright^ NK; NK2, *CD16*^+^*CD56*^dim^*CD57*^−^ NK; NK3, the *CD16*^+^*CD56*^dim^*CD57*^+^ late NK; NRF1, nuclear respiratory factor 1; PCA, principal component analysis; RBCs, red blood cells; RT-qPCR, quantitative reverse transcription polymerase chain reaction; scATAC-seq, single-cell assay for transposase-accessible chromatin sequencing; scRNA-seq, single-cell RNA sequencing; scTCR/BCR-seq, single-cell paired TCR/BCR sequencing; TCs, T cells; TFs, transcription factors; TNF, tumor necrosis factor; IL, interleukin; T-mito, mitotic T cells; t-SNE, t-distributed stochastic neighbor embedding; UMIs, unique molecular identifiers; YA, young healthy adults; YCO, young COVID-19 onset patients; YCR, young recovered COVID-19 patients; YH, young healthy.
