# Supplementary material for: A human circulating immune cell landscape in aging and COVID-19
Source: Protein Cell. 2020 Aug 11;11(10):740–70. doi: 10.1007/s13238-020-00762-2 (PMC7417788; doi:10.1007/s13238-020-00762-2)
Supplement: 13238_2020_762_MOESM1_ESM — Supplementary Material [file 13238_2020_762_moesm1_esm.pdf]

Supplementary Material

Supplementary Figure Legends

Figure S1. Clusters of major immune cell populations in the YA and AA groups derived from scRNA-seq data.
A. t-SNE projections of PBMCs derived from scRNA-seq data in cohort-1.
B. t-SNE plots segregated into YA and AA groups.
C. t-SNE projection of canonical markers, including *CD3E, CD4, CD8A, STMN1, NCAM1, NKG7, CD19, MS4A1, CD14, FCGR3A, HLA-DRA, CD1c, MZB1, CLEC4C, HBB, PF4*.

Figure S2. Clusters of major immune cell populations in the YA and AA groups derived from mass cytometry data.
A. t-SNE projections of PBMCs derived from mass cytometry data in cohort-1.
B. t-SNE plots segregated by YA and AA groups.
C. t-SNE projection of canonical markers, including CD3, CD4, CD8A, CD11C, CD56, CD57, CD19, CD20, CD14, CD16, HLA-DRA, CD123.
D. Heatmap showing mean population expression levels of all markers.

Figure S3. Re-clustering of cell subsets derived from scRNA-seq data in cohort-1.
A. t-SNE projections of $CD3^+$ T cell subsets.
B. t-SNE projections of $CD4^+$ T cell subsets.
C. t-SNE projections of $CD8^+$ T cell subsets.
D. t-SNE projections of NK cell subsets.
E. t-SNE projections of B cell subsets.
F. t-SNE projections of monocyte subsets.
G. t-SNE projections of DC subsets.
H. t-SNE projection of canonical markers in T cell subsets.
I. t-SNE projection of canonical markers in $CD4^+$ T cell subsets.
J. t-SNE projection of canonical markers in $CD8^+$ T cell subsets.
K. t-SNE projection of canonical markers in NK cell subsets.
L. t-SNE projection of canonical markers in B cell subsets.
M. t-SNE projection of canonical markers in monocyte subsets.
N. t-SNE projection of canonical markers in DC subsets.

Figure S4. Re-clusters of cell subsets derived from mass cytometry data in cohort-1.
A. t-SNE projections of T cell subsets.
B. t-SNE projections of NK cell subsets.
C. t-SNE projections of B cell subsets.
D. t-SNE projections of monocyte subsets.
E. t-SNE projections of DC subsets.
F. t-SNE projection of canonical markers in T cell subsets.
G. t-SNE projection of canonical markers in NK cell subsets.
H. t-SNE projection of canonical markers in B cell subsets.
I. t-SNE projection of canonical markers in monocyte subsets.
J. t-SNE projection of canonical markers in DC subsets.
K. Heatmap showing mean population expression levels of all markers.

Figure S5. Changes in cell proportions during PBMC aging.
A. Percentage of major immune cells in PBMCs in YA (n = 8) and AA (n = 8) from scRNA-seq data.
B. Percentage of major immune cells in $CD45^+$ cells in YA (n = 5) and AA (n = 5) from mass cytometry data.
C. Bar chart of the relative percentage of NK subsets from scRNA-seq data.
D. Bar chart of the relative percentage of NK subsets from mass cytometry data.
E. Bar chart of the relative percentage of BC subsets from scRNA-seq data.
F. Bar chart of the relative percentage of BC subsets from mass cytometry data.
G. Bar chart of the relative percentage of monocyte subsets from scRNA-seq data.
H. Bar chart of the relative percentage of monocyte subsets from mass cytometry data.
I. Percentage of cell subsets in PBMCs in YA (n = 8) and AA (n = 8) from scRNA-seq data.
J. Percentage of cell subsets in PBMCs in YA (n = 5) and AA (n = 5) from mass cytometry data.
P values are based on two-tailed Mann-Whitney-Wilcoxon tests between groups.

Figure S6. Changes in transcriptional profiles of T cells and NK cells during aging.
A. UpSet plot showing integrated comparative analysis of downregulated DEGs in major immune cell populations between YA and AA groups. Downregulated DEGs: downregulated in AA, upregulated in YA.
B. Representative GO terms and pathways enriched in downregulated DEGs based on functional enrichment analysis in major immune cell populations. *P* value was derived by a hypergeometric test.
C. Representative GO terms and pathways enriched in downregulated DEGs based on functional enrichment analysis in $CD4^+$ T cells. *P* value was derived by a hypergeometric test.
D. UpSet plot showing integrated comparative analysis of upregulated DEGs in $CD8^+$ T cells between YA and AA groups. Upregulated DEGs: upregulated in AA, downregulated in YA.
E. Representative GO terms and pathways enriched in upregulated DEGs based on functional enrichment analysis in $CD8^+$ T cells. *P* value was derived by a hypergeometric test.
F. Representative GO terms and pathways enriched in downregulated DEGs based on functional enrichment analysis in $CD8^+$ T cells. *P* value was derived by a hypergeometric test.
G. Volcano plot showing DEGs of T-mito between YA and AA groups. *P* values were calculated using a paired, two-sided Wilcoxon test and FDR was corrected using the Benjamini-Hochberg procedure.
H. Dot plot showing enriched pathways of upregulated and downregulated DEGs in T-mito cluster during aging. *P* value was derived by a hypergeometric test.
I. Representative GO terms and pathways enriched in upregulated DEGs based on functional enrichment analysis in NK cells. *P* value was derived by a hypergeometric test.
J. Representative GO terms and pathways enriched in downregulated DEGs based on functional enrichment analysis in NK cells. *P* value was derived by a hypergeometric test.

Figure S7. Changes in transcriptional profiles of B cells, monocytes and DCs during aging.

A. UpSet plot showing the integrated comparative analysis of upregulated DEGs in B cell subsets between YA and AA groups. Upregulated DEGs: upregulated in AA, downregulated in YA.
B. Representative GO terms and pathways enriched in upregulated DEGs based on functional enrichment analysis in B cell subsets. *P* value was derived by a hypergeometric test.
C. Representative GO terms and pathways enriched in downregulated DEGs based on functional enrichment analysis in B cell subsets. *P* value was derived by a hypergeometric test.
D. Representative GO terms and pathways enriched in downregulated DEGs based on functional enrichment analysis in monocyte subsets. *P* value was derived by a hypergeometric test.
E. UpSet plot showing the integrated comparative analysis of upregulated DEGs in DC subsets between YA and AA groups. Upregulated DEGs: upregulated in AA, downregulated in YA.
F. Representative GO terms and pathways enriched in upregulated DEGs based on functional enrichment analysis in DC subsets. *P* value was derived by a hypergeometric test.
G. UpSet plot showing the integrated comparative analysis of downregulated DEGs in DC subsets between YA and AA group. Downregulated DEGs: downregulated in AA, upregulated in YA.
H. Representative GO terms and pathways enriched in downregulated DEGs based on functional enrichment analysis in DC subsets. *P* value was derived by a hypergeometric test.
I. Violin plots showing the distribution of normalized expression levels of selected aging-associated genes in DC cluster (*CASP4* and *IFITM3* in all DC, *AHR* in cDC2, *MALAT1* in pDC) between YA and AA groups.
J. Heatmap showing biased DEGs within the cDC2-A and cDC2-B cluster.
K. Violin plots showing the distribution of normalized expression levels of selected aging-associated genes in cDC2-A and cDC2-B cluster.

Figure S8. Changes in chromosomal accessibility during aging.
A. t-SNE projection of canonical markers, including *CD3E, CD4, CD8A, NCAM1, NKG7, CD19, CD20, CD14, FCGR3A, ITGA2B.*
B. t-SNE projections of NK cells and T cells derived from scATAC-seq data.
C. t-SNE projections of B cells derived from scATAC-seq data.
D. t-SNE projections of monocytes and DCs derived from scATAC-seq data.
E. Venn diagram showing the integrated comparative analysis of downregulated differentially expressed transcription factors (DETs) in $CD4^+$ T cell subsets between YA and AA group. Downregulated DETs: downregulated in AA, upregulated in YA.
F. Venn diagram showing the integrated comparative analysis of downregulated DETs in $CD8^+$ T cell subsets between YA and AA group. Downregulated DETs: downregulated in AA, upregulated in YA.
G. Venn diagram showing the integrated comparative analysis of downregulated DETs in NK subsets between YA and AA group. Downregulated DETs: downregulated in AA, upregulated in YA.
H. Mean scATAC-seq coverage at *NRF1* loci in all cells.
I. Mean scATAC-seq coverage at *ELK4* loci in Memory B cells.
J. Mean scATAC-seq coverage at *GZMB* loci in CD8 CTL.
K. Mean scATAC-seq coverage at *IL1B* loci in CD14 MC.

L. Mean scATAC-seq coverage at *TNF* loci in CD16 MC.
M. Mean scATAC-seq coverage at *CXCL8* loci in CD16 MC.

Figure S9. Abnormal TCR and BCR repertoire during aging.
A. Bar plot showing specific TRA usage in the YA and AA groups.
B. Bar plot showing specific TRB usage in the YA and AA groups.
C. Bar plot showing specific IGH usage in the YA and AA groups.
D. Bar plot showing specific IGK usage in the YA and AA groups.
E. Bar plot showing specific IGL usage in the YA and AA groups.
F. Bar plot showing the relative percentage of each isotype in the YA and AA groups.
G. Bar plot showing the ratio of (*IGHA* + *IGHG*) to (*IGHD* + *IGHM*) among the YA and AA groups (n = 8/group). *P* values are based on two-tailed Mann-Whitney-Wilcoxon tests between groups.
H. Chord diagram showing the pairing of V and J segments within TRB subset from the YA group. Chord widths represent the proportion of sequences with a given V (colored) and J (gray) segment pairing.
I. Chord diagram showing the pairing of V and J segments within IGH subset from the YA group. Chord widths represent the proportion of sequences with a given V (colored) and J (gray) segment pairing.

Figure S10. Clusters of major immune cell populations derived from mass cytometry data in cohort-2.
A. t-SNE projections of PBMCs.
B. t-SNE plots segregated by YH, AH, YCO and ACO groups.
C. t-SNE projection of canonical markers, including CD3, CD4, CD8A, CD11C, CD56, CD57, CD19, CD20, CD14, CD16, HLA-DRA, CD123.

Figure S11. Re-Clustering of cell subsets derived from mass cytometry data in cohort-2.
A. t-SNE projections of T cell subsets.
B. t-SNE projections of NK cell subsets.
C. t-SNE projections of B cell subsets.
D. t-SNE projections of monocyte subsets.
E. t-SNE projections of DC subsets derived.
F. t-SNE projection of canonical markers in T cell subsets.
G. t-SNE projection of canonical markers in NK cell subsets.
H. t-SNE projection of canonical markers in B cell subsets.
I. t-SNE projection of canonical markers in monocytes subsets.
J. t-SNE projection of canonical markers in DC subsets.
K. Heatmap showing mean population expression levels of all markers.

Figure S12. Poor outcome upon COVID-19 infection is associated with imbalanced cellular aging.
A. Bar chart of the relative percentage of all cell subsets derived from mass cytometry data in cohort-2. HC includes YH (n = 2) and AH (n = 2); CO includes YCO (n = 2) and ACO (n = 2).
B. Bar chart of the relative percentage of T cell subsets derived from mass cytometry data in cohort-2.
C. Percentage of CD8 Naive T cells in $CD45^+$ cells derived from mass cytometry data between HC (n = 4) and CO (n = 4) groups.

D. Percentage of CD8 CTL in $CD45^+$ cells derived from mass cytometry data between HC (n = 4) and CO (n = 4) groups.
E. Bar chart of the relative percentage of B cells subsets derived from mass cytometry data between HC and CO group.
F. Percentage of memory BC in $CD45^+$ cells between HC (n = 4) and CO (n = 4) groups.
G. Percentage of ASC in $CD45^+$ cells between HC (n = 4) and CO (n = 4) groups.
H. Bar chart of the relative percentage of NK subsets derived from mass cytometry data between HC and CO group.
I. Bar chart of the relative percentage of monocyte subsets derived from mass cytometry data between HC and CO group.
J. Percentage of intermediate monocytes in $CD45^+$ cells between HC (n = 4) and CO (n = 4) groups.
K. Bar chart of the relative percentage of DC subsets derived from mass cytometry data between HC and CO group.
L. Percentage of pDC in $CD45^+$ cells between HC (n = 4) and CO (n = 4) groups.
M. Bar chart of the relative percentage of all cell subsets derived from mass cytometry data between YH, AH and ACO groups.
N. Bar chart of the relative percentage of all cell subsets derived from mass cytometry data between YCO and ACO groups.
O. Bar chart of the relative percentage of T cell subsets derived from mass cytometry data between YCO and ACO groups.
P. Bar chart of the relative percentage of NK subsets derived from mass cytometry data between YCO and ACO groups.
Q. Bar chart of the relative percentage of BC subsets derived from mass cytometry data between YCO and ACO groups.
R. Bar chart of the relative percentage of monocyte subsets derived from mass cytometry data between YCO and ACO groups.
S. Bar chart of the relative percentage of DC subsets derived from mass cytometry data between YCO and ACO groups.
P values are based on two-tailed Mann-Whitney-Wilcoxon tests between groups.

Figure S13. Clusters of major immune cell populations derived from scRNA-seq data in cohort-3.
A. t-SNE projections of PBMCs.
B. t-SNE plots segregated by YH, AH, YCR and ACR groups.
C. t-SNE projection of canonical markers, including *CD3E, CD4, CD8A, STMN1, CCR7, FOXP3, AQP3, CCR6, GZMK, GZMB, NCAM1, B3GAT1, FCGR3A, HBB, PF4, CD19, MS4A1, IL4R, CD27, MZB1, ITGAX, CD14, CLEC9A, CD1c, CLEC4C.*

Figure S14. Aging and SARS-CoV-2 infection are characterized by similar hyper-inflammatory states.
A. Heatmaps showing scaled expression of discriminative gene sets for each cell type and cell subset in cohort-3. Color scheme is based on z-score distribution from -3 (purple) to 3 (yellow).
B. Violin plot showing the distribution of normalized expression levels of *NFATC1* in CD4 Naive cells cluster between YH and AH groups.
C. Violin plot showing the distribution of normalized expression levels of *ITGB1* in CD4 Naive cells cluster between YH and AH groups.
D. Violin plot showing the distribution of normalized expression levels of *PPIB* in CD4 Naive cells cluster between YH and AH groups.

E. Violin plot showing the distribution of normalized expression levels of *DPP4* in CD4 Naive cells cluster between YH and AH groups.
F. Bar charts of the relative percentage of all cell subsets between YH, AH and ACR groups (left), YCR and ACR groups (right).

Figure S15. Cell-cell interactions associated with disease recovering events.
A-B. Dot plots showing specific differentially expressed ligand-receptor interactions between YCR and ACR groups based on Log_2 FC across ligands. Ligand-receptor interactions are shown for TC-MC interactions (A), BC-MC interactions (B). GO terms associated with the functions of the ligands and receptors are labeled to the left.

Supplementary Table Legends

Table S1. Sample information of single-cell PBMCs collected from three separate cohorts for scRNA-seq, CyTOF, scATAC-seq and scTCR/BCR-seq.
Table S2. Antibodies used in CyTOF analysis.
Table S3. Cell clustering strategy in scRNA-seq, CyTOF, scATAC-seq.
Table S4. DEGs in five major immune cells between AA and YA groups.
Table S5. DEGs in $CD4^+$ TC, $CD8^+$ TC and T-mito subset between AA and YA groups.
Table S6. DEGs in $CD4^+$ TC subpopulation between AA and YA groups.
Table S7. DEGs in $CD8^+$ TC subpopulation between AA and YA groups.
Table S8. DEGs in NK subpopulation between AA and YA groups.
Table S9. DEGs in BC subpopulation between AA and YA groups.
Table S10. DEGs in MC subpopulation between AA and YA groups.
Table S11. DEGs in DC subpopulation between AA and YA groups.
Table S12. DEGs in clonal TC and BC between AA and YA groups.
Table S13. DEGs in TC between AH and YH groups (A), YCR and YH groups (B), ACR and AH groups (C), ACR and YCR groups (D).
Table S14. DEGs in MC between AH and YH groups (A), YCR and YH groups (B), ACR and AH groups (C), ACR and YCR groups (D).

Figure. S1

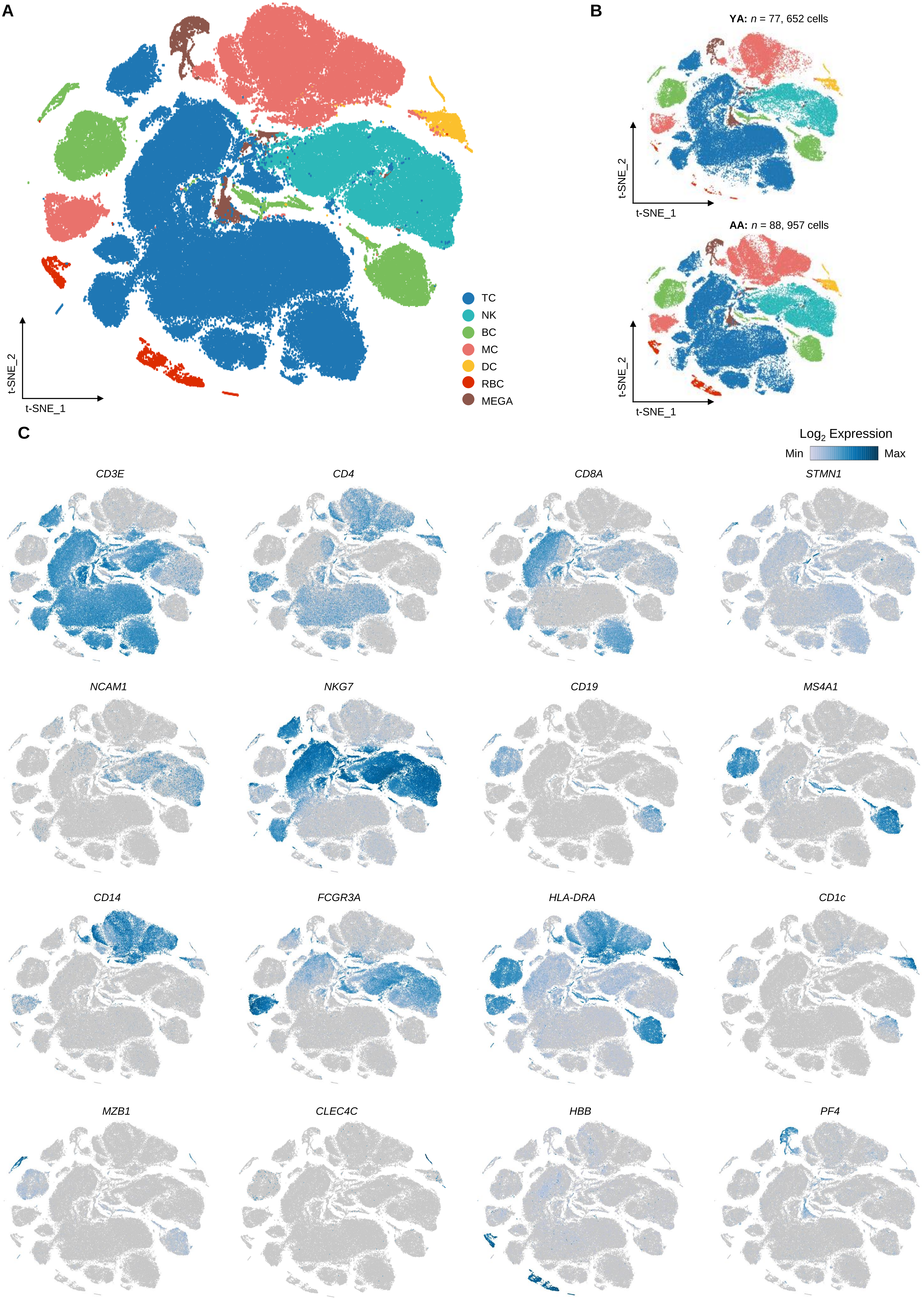
A
t-SNE_2
t-SNE_1
TC
NK
BC
MC
DC
RBC
MEGA
B
YA: n = 77, 652 cells
t-SNE_2
t-SNE_1
AA: n = 88, 957 cells
t-SNE_2
t-SNE_1
C
Log2 Expression
Min
Max
CD3E
CD4
CD8A
STMN1
NCAM1
NKG7
CD19
MS4A1
CD14
FCGR3A
HLA-DRA
CD1c
MZB1
CLEC4C
HBB
PF4

Figure. S2

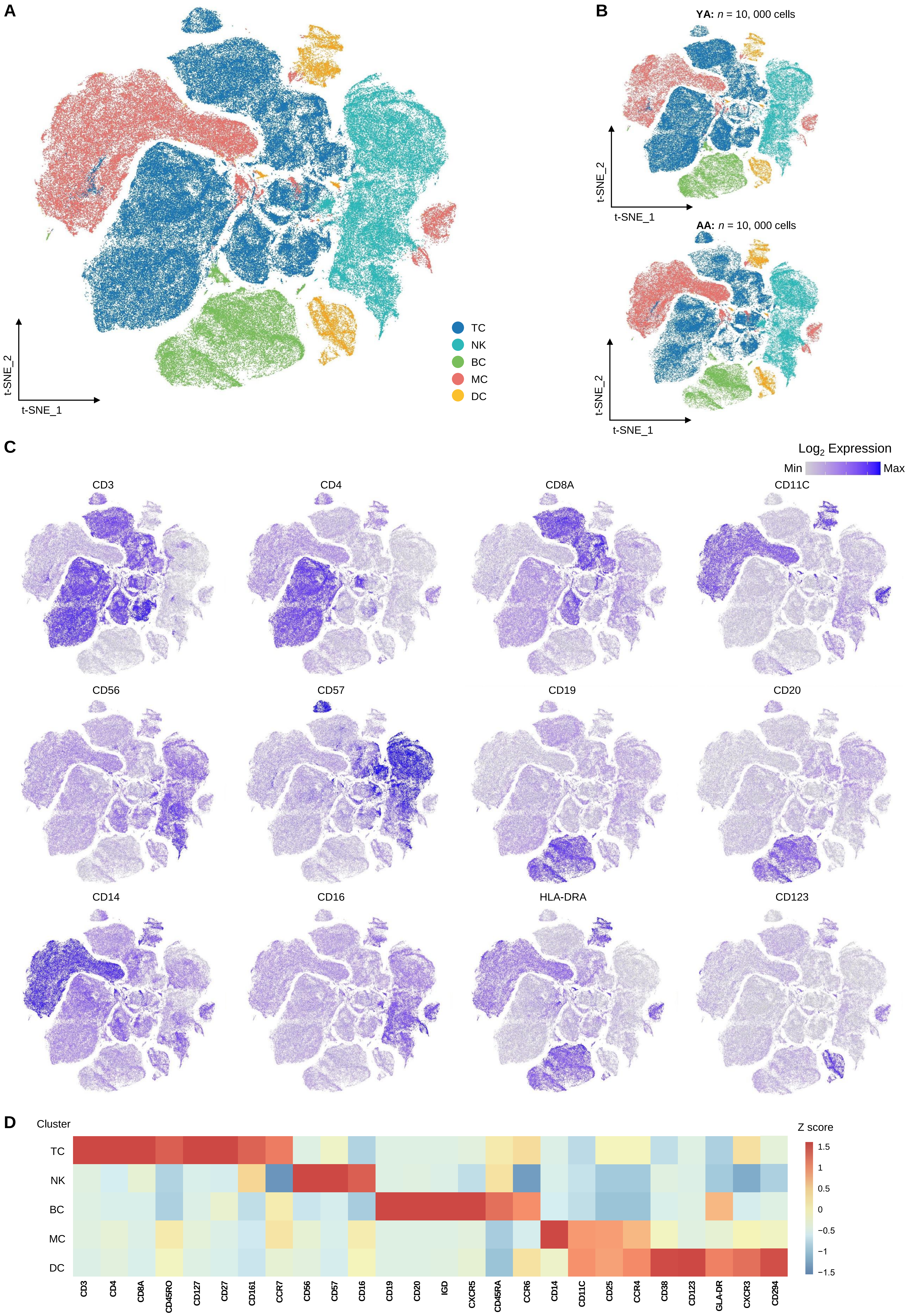
A
B
YA: n = 10, 000 cells
AA: n = 10, 000 cells
t-SNE_1
t-SNE_2
TC
NK
BC
MC
DC
C
Log2 Expression
Min
Max
CD3
CD4
CD8A
CD11C
CD56
CD57
CD19
CD20
CD14
CD16
HLA-DRA
CD123
D
Cluster
Z score
1.5
1
0.5
0
−0.5
−1
−1.5
TC
NK
BC
MC
DC
CD3
CD4
CD8A
CD45RO
CD127
CD27
CD161
CCR7
CD56
CD57
CD16
CD19
CD20
IGD
CXCR5
CD45RA
CCR6
CD14
CD11C
CD25
CCR4
CD38
CD123
GLA-DR
CXCR3
CD294

Figure. S3

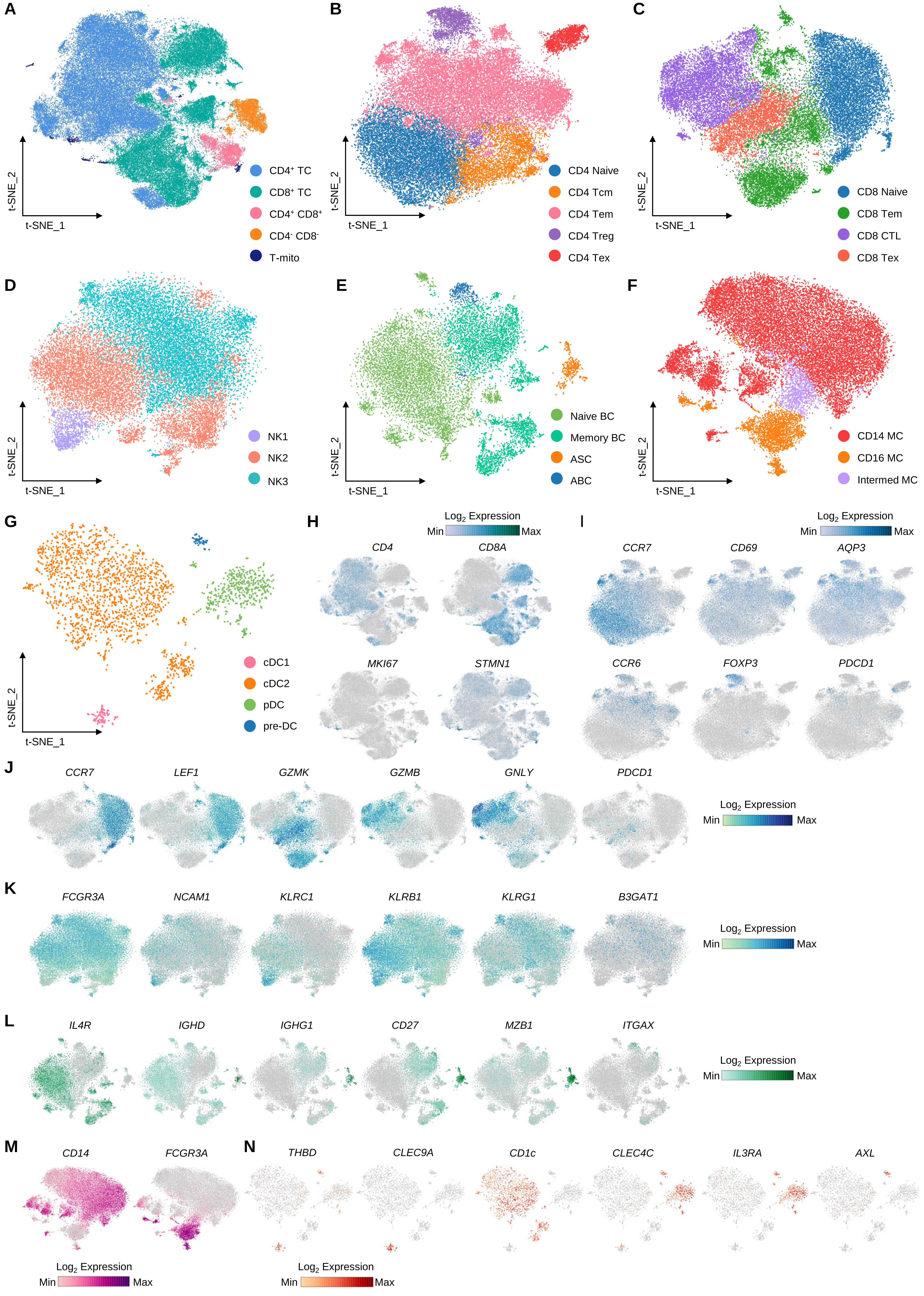
A
t-SNE_2
t-SNE_1
CD4+ TC
CD8+ TC
CD4+ CD8+
CD4- CD8-
T-mito
B
CD4 Naive
CD4 Tcm
CD4 Tem
CD4 Treg
CD4 Tex
C
CD8 Naive
CD8 Tem
CD8 CTL
CD8 Tex
D
NK1
NK2
NK3
E
Naive BC
Memory BC
ASC
ABC
F
CD14 MC
CD16 MC
Intermed MC
G
cDC1
cDC2
pDC
pre-DC
H
Log2 Expression
Min
Max
CD4
CD8A
MKI67
STMN1
I
CCR7
CD69
AQP3
CCR6
FOXP3
PDCD1
J
CCR7
LEF1
GZMK
GZMB
GNLY
PDCD1
K
FCGR3A
NCAM1
KLRC1
KLRB1
KLRG1
B3GAT1
L
IL4R
IGHD
IGHG1
CD27
MZB1
ITGAX
M
CD14
FCGR3A
N
THBD
CLEC9A
CD1c
CLEC4C
IL3RA
AXL

Figure. S4

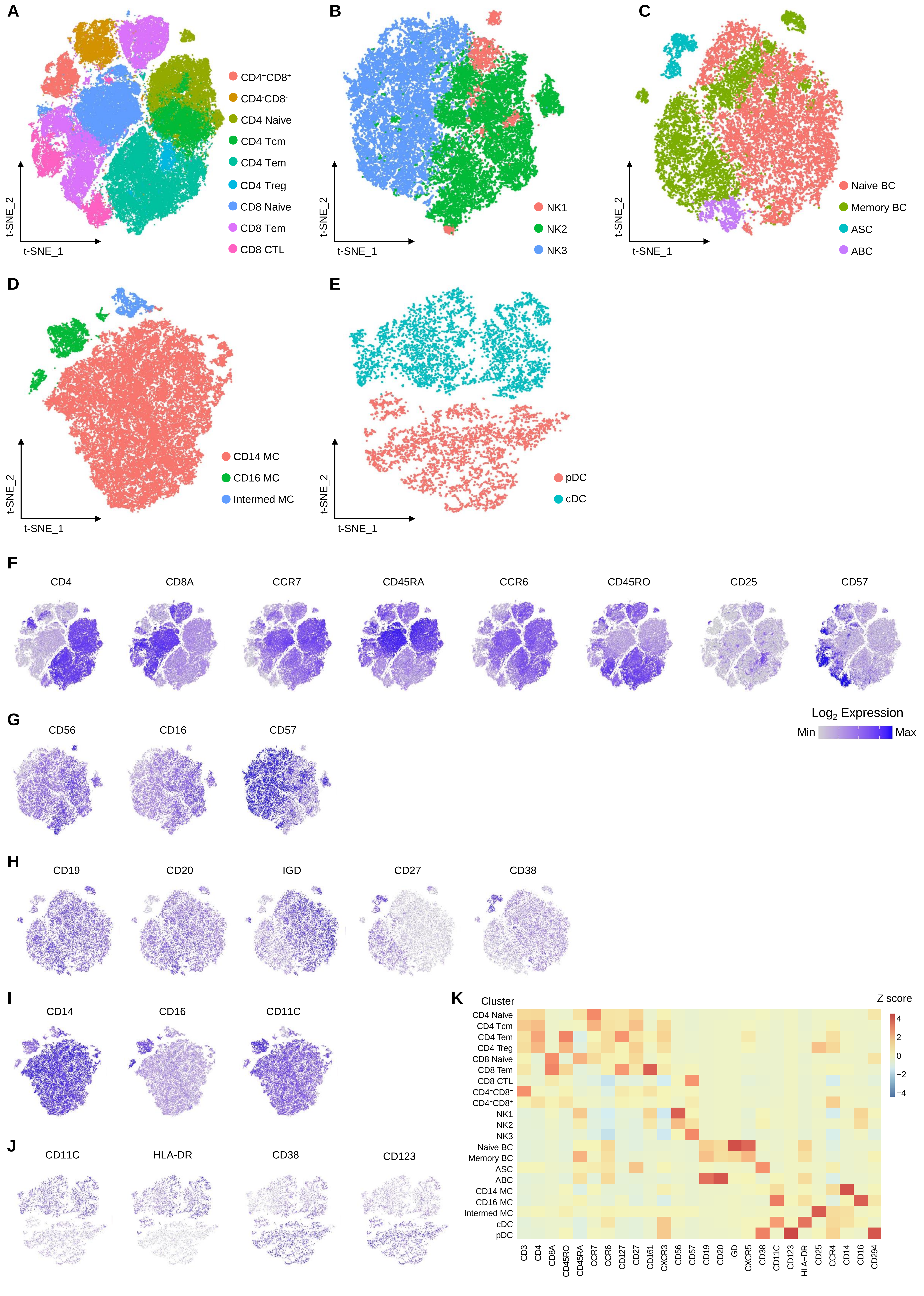
A
CD4+CD8+
CD4-CD8-
CD4 Naive
CD4 Tcm
CD4 Tem
CD4 Treg
CD8 Naive
CD8 Tem
CD8 CTL
t-SNE_2
t-SNE_1
B
NK1
NK2
NK3
C
Naive BC
Memory BC
ASC
ABC
D
CD14 MC
CD16 MC
Intermed MC
E
pDC
cDC
F
CD4
CD8A
CCR7
CD45RA
CCR6
CD45RO
CD25
CD57
G
CD56
CD16
CD57
Log2 Expression
Min
Max
H
CD19
CD20
IGD
CD27
CD38
I
CD14
CD16
CD11C
J
CD11C
HLA-DR
CD38
CD123
K
Cluster
Z score
4
2
0
−2
−4
CD4 Naive
CD4 Tcm
CD4 Tem
CD4 Treg
CD8 Naive
CD8 Tem
CD8 CTL
CD4−CD8−
CD4+CD8+
NK1
NK2
NK3
Naive BC
Memory BC
ASC
ABC
CD14 MC
CD16 MC
Intermed MC
cDC
pDC
CD3
CD4
CD8A
CD45RO
CD45RA
CCR7
CCR6
CD127
CD27
CD161
CXCR3
CD56
CD57
CD19
CD20
IGD
CXCR5
CD38
CD11C
CD123
HLA−DR
CD25
CCR4
CD14
CD16
CD294

Figure. S5

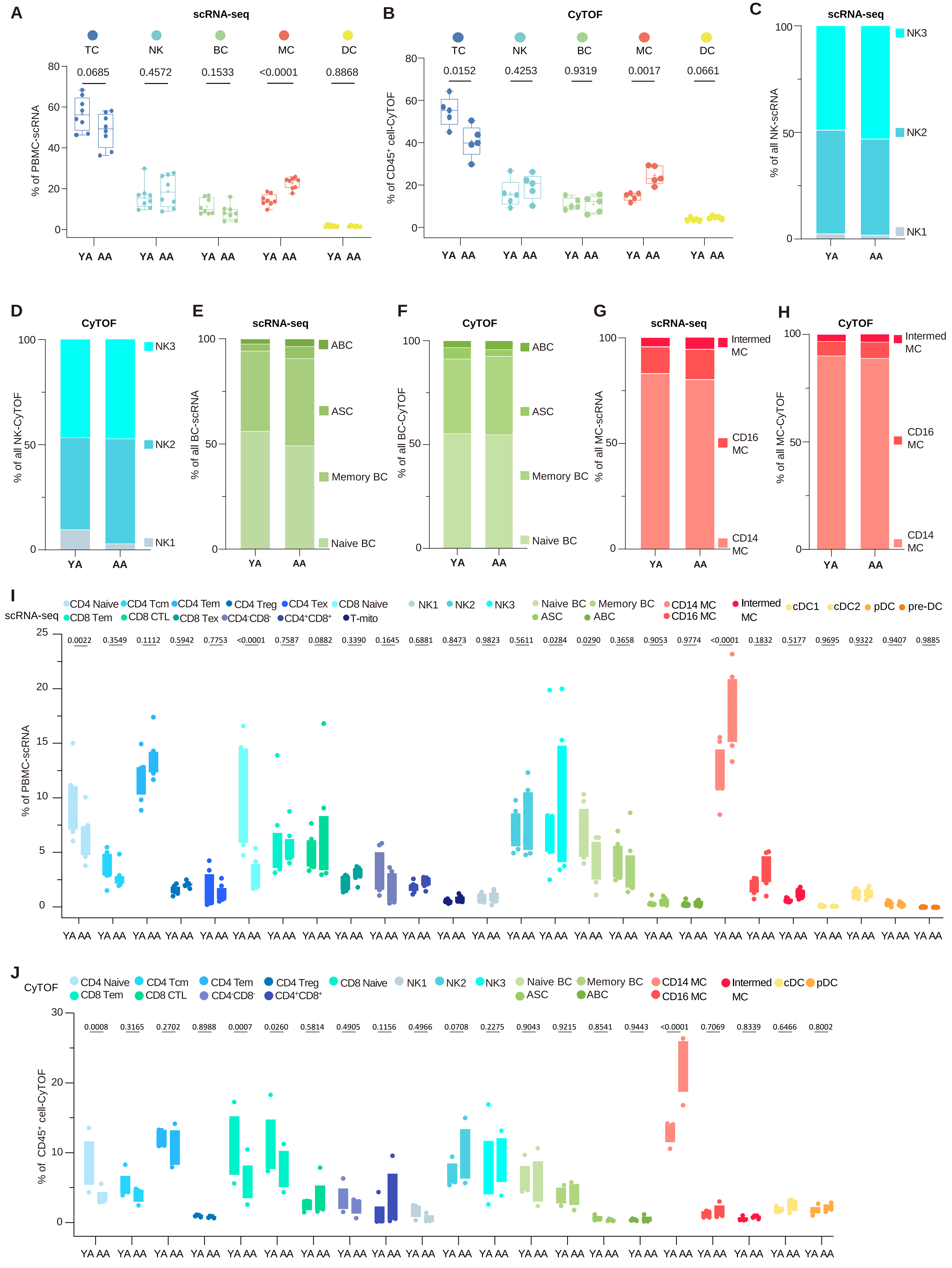

A
scRNA-seq
TC
NK
BC
MC
DC
0.0685
0.4572
0.1533
<0.0001
0.8868
% of PBMC-scRNA
YA AA
B
CyTOF
TC
NK
BC
MC
DC
0.0152
0.4253
0.9319
0.0017
0.0661
% of CD45⁺ cell-CyTOF
YA AA
C
scRNA-seq
NK3
NK2
NK1
% of all NK-scRNA
YA
AA
D
CyTOF
NK3
NK2
NK1
% of all NK-CyTOF
E
scRNA-seq
ABC
ASC
Memory BC
Naive BC
% of all BC-scRNA
F
CyTOF
ABC
ASC
Memory BC
Naive BC
% of all BC-CyTOF
G
scRNA-seq
Intermed MC
CD16 MC
CD14 MC
% of all MC-scRNA
H
CyTOF
Intermed MC
CD16 MC
CD14 MC
% of all MC-CyTOF
I
scRNA-seq
CD4 Naive
CD4 Tcm
CD4 Tem
CD4 Treg
CD4 Tex
CD8 Naive
CD8 Tem
CD8 CTL
CD8 Tex
CD4⁻CD8⁻
CD4⁺CD8⁺
T-mito
NK1
NK2
NK3
Naive BC
Memory BC
ASC
ABC
CD14 MC
CD16 MC
Intermed MC
cDC1
cDC2
pDC
pre-DC
0.0022 0.3549 0.1112 0.5942 0.7753 <0.0001 0.7587 0.0882 0.3390 0.1645 0.6881 0.8473 0.9823 0.5611 0.0284 0.0290 0.3658 0.9053 0.9774 <0.0001 0.1832 0.5177 0.9695 0.9322 0.9407 0.9885
% of PBMC-scRNA
J
CyTOF
CD4 Naive
CD4 Tcm
CD4 Tem
CD4 Treg
CD8 Naive
CD8 Tem
CD8 CTL
CD4⁻CD8⁻
CD4⁺CD8⁺
NK1
NK2
NK3
Naive BC
Memory BC
ASC
ABC
CD14 MC
CD16 MC
Intermed MC
cDC
pDC
0.0008 0.3165 0.2702 0.8988 0.0007 0.0260 0.5814 0.4905 0.1156 0.4966 0.0708 0.2275 0.9043 0.9215 0.8541 0.9443 <0.0001 0.7069 0.8339 0.6466 0.8002
% of CD45⁺ cell-CyTOF

Figure. S6

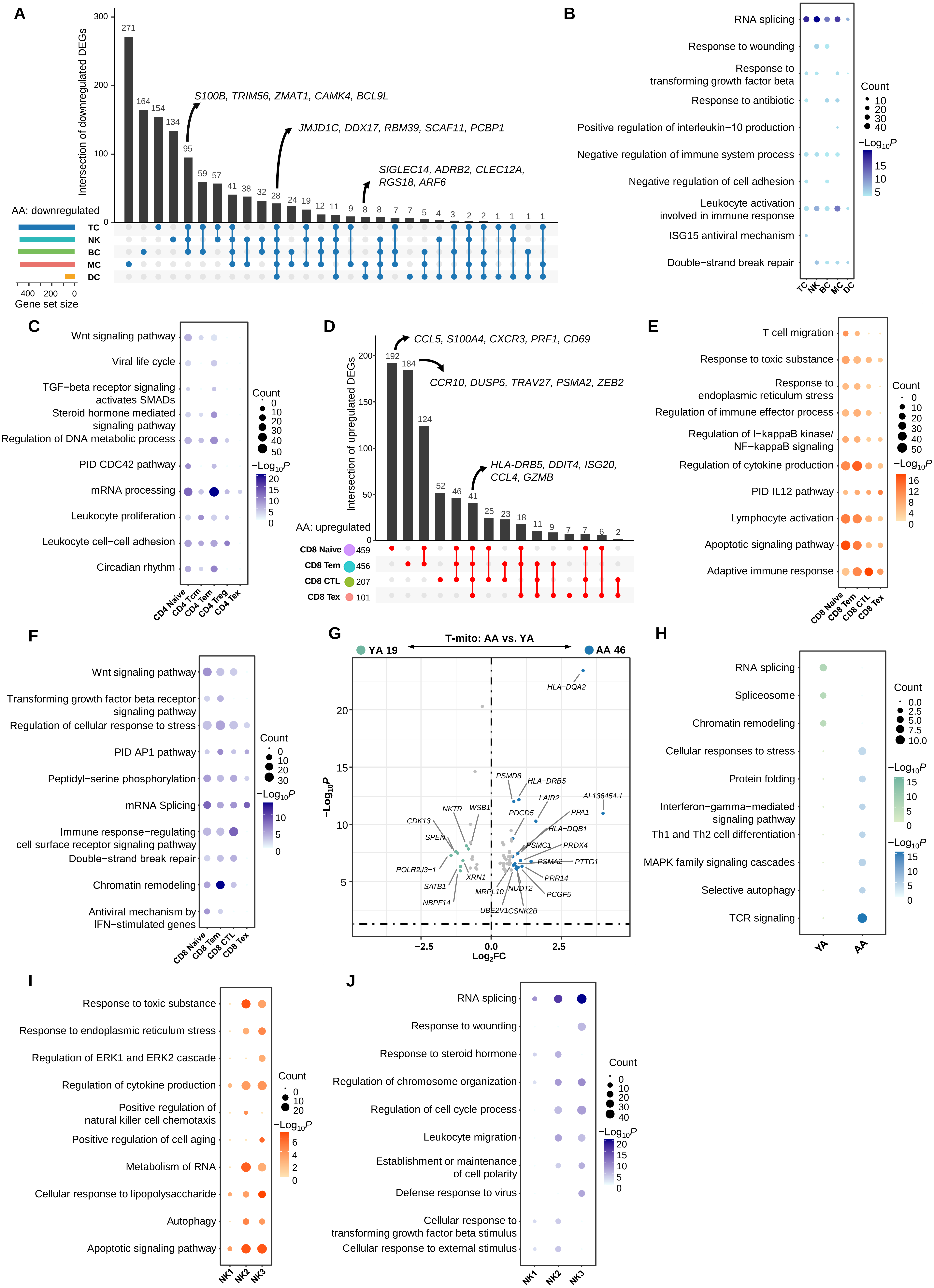
A
AA: downregulated
Intersection of downregulated DEGs
S100B, TRIM56, ZMAT1, CAMK4, BCL9L
JMJD1C, DDX17, RBM39, SCAF11, PCBP1
SIGLEC14, ADRB2, CLEC12A, RGS18, ARF6
TC
NK
BC
MC
DC
Gene set size
B
RNA splicing
Response to wounding
Response to transforming growth factor beta
Response to antibiotic
Positive regulation of interleukin−10 production
Negative regulation of immune system process
Negative regulation of cell adhesion
Leukocyte activation involved in immune response
ISG15 antiviral mechanism
Double−strand break repair
Count
C
Wnt signaling pathway
Viral life cycle
TGF−beta receptor signaling activates SMADs
Steroid hormone mediated signaling pathway
Regulation of DNA metabolic process
PID CDC42 pathway
mRNA processing
Leukocyte proliferation
Leukocyte cell−cell adhesion
Circadian rhythm
CD4 Naive
CD4 Tcm
CD4 Tem
CD4 Treg
CD4 Tex
D
AA: upregulated
Intersection of upregulated DEGs
CCL5, S100A4, CXCR3, PRF1, CD69
CCR10, DUSP5, TRAV27, PSMA2, ZEB2
HLA-DRB5, DDIT4, ISG20, CCL4, GZMB
CD8 Naive 459
CD8 Tem 456
CD8 CTL 207
CD8 Tex 101
E
T cell migration
Response to toxic substance
Response to endoplasmic reticulum stress
Regulation of immune effector process
Regulation of I−kappaB kinase/NF−kappaB signaling
Regulation of cytokine production
PID IL12 pathway
Lymphocyte activation
Apoptotic signaling pathway
Adaptive immune response
CD8 Naive
CD8 Tem
CD8 CTL
CD8 Tex
F
Wnt signaling pathway
Transforming growth factor beta receptor signaling pathway
Regulation of cellular response to stress
PID AP1 pathway
Peptidyl−serine phosphorylation
mRNA Splicing
Immune response−regulating cell surface receptor signaling pathway
Double−strand break repair
Chromatin remodeling
Antiviral mechanism by IFN−stimulated genes
G
T-mito: AA vs. YA
YA 19
AA 46
Log2FC
HLA−DQA2
PSMD8
HLA−DRB5
LAIR2
AL136454.1
NKTR
WSB1
PDCD5
PPA1
CDK13
HLA−DQB1
SPEN
PSMC1
PRDX4
PSMA2
PTTG1
POLR2J3−1
XRN1
PRR14
SATB1
MRPL10
NUDT2
PCGF5
NBPF14
UBE2V1
CSNK2B
H
RNA splicing
Spliceosome
Chromatin remodeling
Cellular responses to stress
Protein folding
Interferon−gamma−mediated signaling pathway
Th1 and Th2 cell differentiation
MAPK family signaling cascades
Selective autophagy
TCR signaling
YA
AA
I
Response to toxic substance
Response to endoplasmic reticulum stress
Regulation of ERK1 and ERK2 cascade
Regulation of cytokine production
Positive regulation of natural killer cell chemotaxis
Positive regulation of cell aging
Metabolism of RNA
Cellular response to lipopolysaccharide
Autophagy
Apoptotic signaling pathway
NK1
NK2
NK3
J
RNA splicing
Response to wounding
Response to steroid hormone
Regulation of chromosome organization
Regulation of cell cycle process
Leukocyte migration
Establishment or maintenance of cell polarity
Defense response to virus
Cellular response to transforming growth factor beta stimulus
Cellular response to external stimulus

Figure. S7

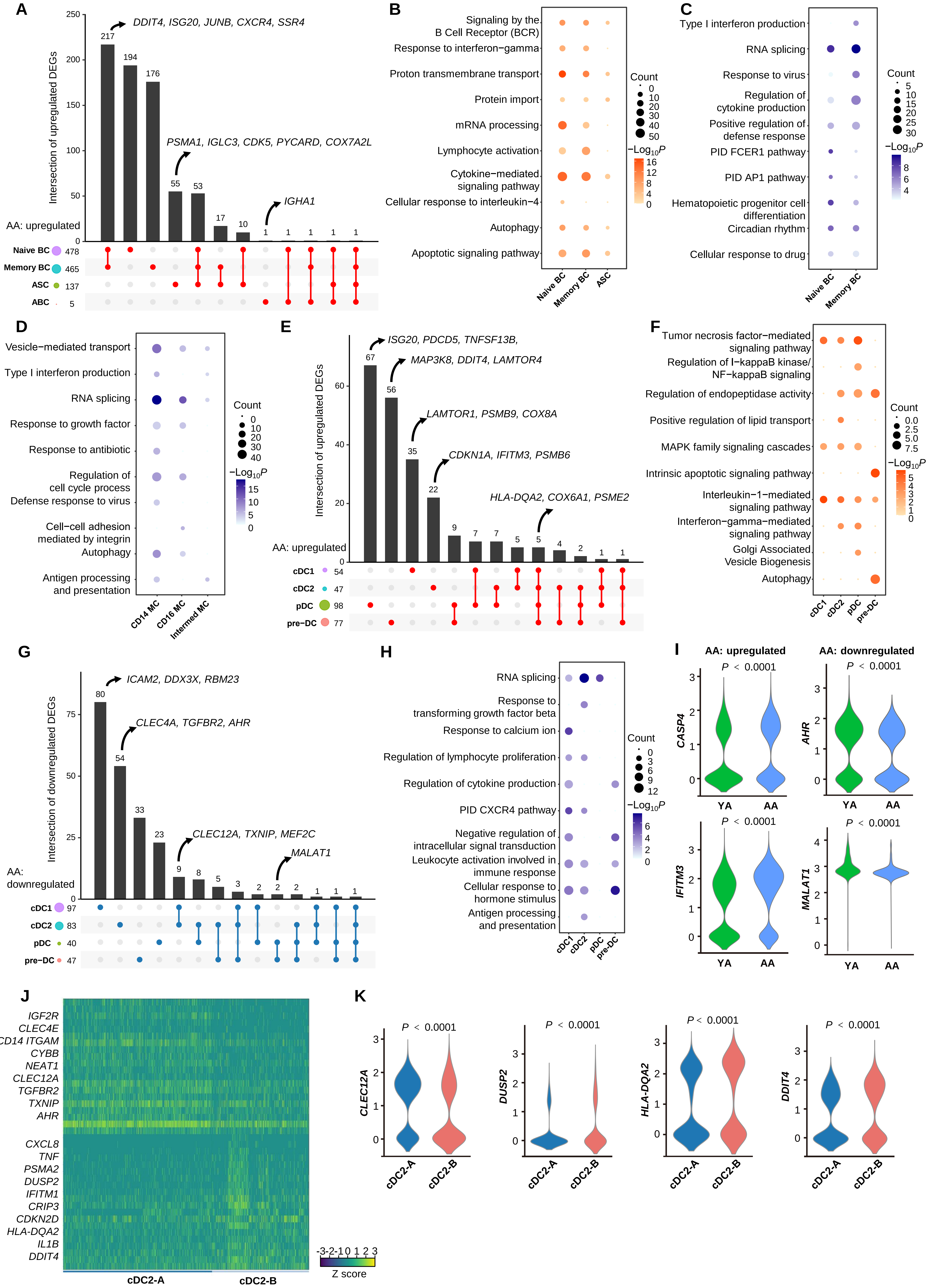
A
B
C
D
E
F
G
H
I
J
K
AA: upregulated
AA: downregulated
Intersection of upregulated DEGs
Intersection of downregulated DEGs
DDIT4, ISG20, JUNB, CXCR4, SSR4
PSMA1, IGLC3, CDK5, PYCARD, COX7A2L
IGHA1
Naive BC
Memory BC
ASC
ABC
Signaling by the B Cell Receptor (BCR)
Response to interferon−gamma
Proton transmembrane transport
Protein import
mRNA processing
Lymphocyte activation
Cytokine−mediated signaling pathway
Cellular response to interleukin−4
Autophagy
Apoptotic signaling pathway
Count
Type I interferon production
RNA splicing
Response to virus
Regulation of cytokine production
Positive regulation of defense response
PID FCER1 pathway
PID AP1 pathway
Hematopoietic progenitor cell differentiation
Circadian rhythm
Cellular response to drug
Vesicle−mediated transport
Response to growth factor
Response to antibiotic
Regulation of cell cycle process
Defense response to virus
Cell−cell adhesion mediated by integrin
Antigen processing and presentation
CD14 MC
CD16 MC
Intermed MC
ISG20, PDCD5, TNFSF13B,
MAP3K8, DDIT4, LAMTOR4
LAMTOR1, PSMB9, COX8A
CDKN1A, IFITM3, PSMB6
HLA-DQA2, COX6A1, PSME2
cDC1
cDC2
pDC
pre−DC
Tumor necrosis factor−mediated signaling pathway
Regulation of I−kappaB kinase/ NF−kappaB signaling
Regulation of endopeptidase activity
Positive regulation of lipid transport
MAPK family signaling cascades
Intrinsic apoptotic signaling pathway
Interleukin−1−mediated signaling pathway
Interferon−gamma−mediated signaling pathway
Golgi Associated Vesicle Biogenesis
ICAM2, DDX3X, RBM23
CLEC4A, TGFBR2, AHR
CLEC12A, TXNIP, MEF2C
MALAT1
Response to transforming growth factor beta
Response to calcium ion
Regulation of lymphocyte proliferation
PID CXCR4 pathway
Negative regulation of intracellular signal transduction
Leukocyte activation involved in immune response
Cellular response to hormone stimulus
P < 0.0001
CASP4
AHR
IFITM3
MALAT1
YA
AA
IGF2R
CLEC4E
CD14 ITGAM
CYBB
NEAT1
CLEC12A
TGFBR2
TXNIP
AHR
CXCL8
TNF
PSMA2
DUSP2
IFITM1
CRIP3
CDKN2D
HLA-DQA2
IL1B
DDIT4
Z score
cDC2-A
cDC2-B
CLEC12A
DUSP2
HLA-DQA2
DDIT4

Figure. S8

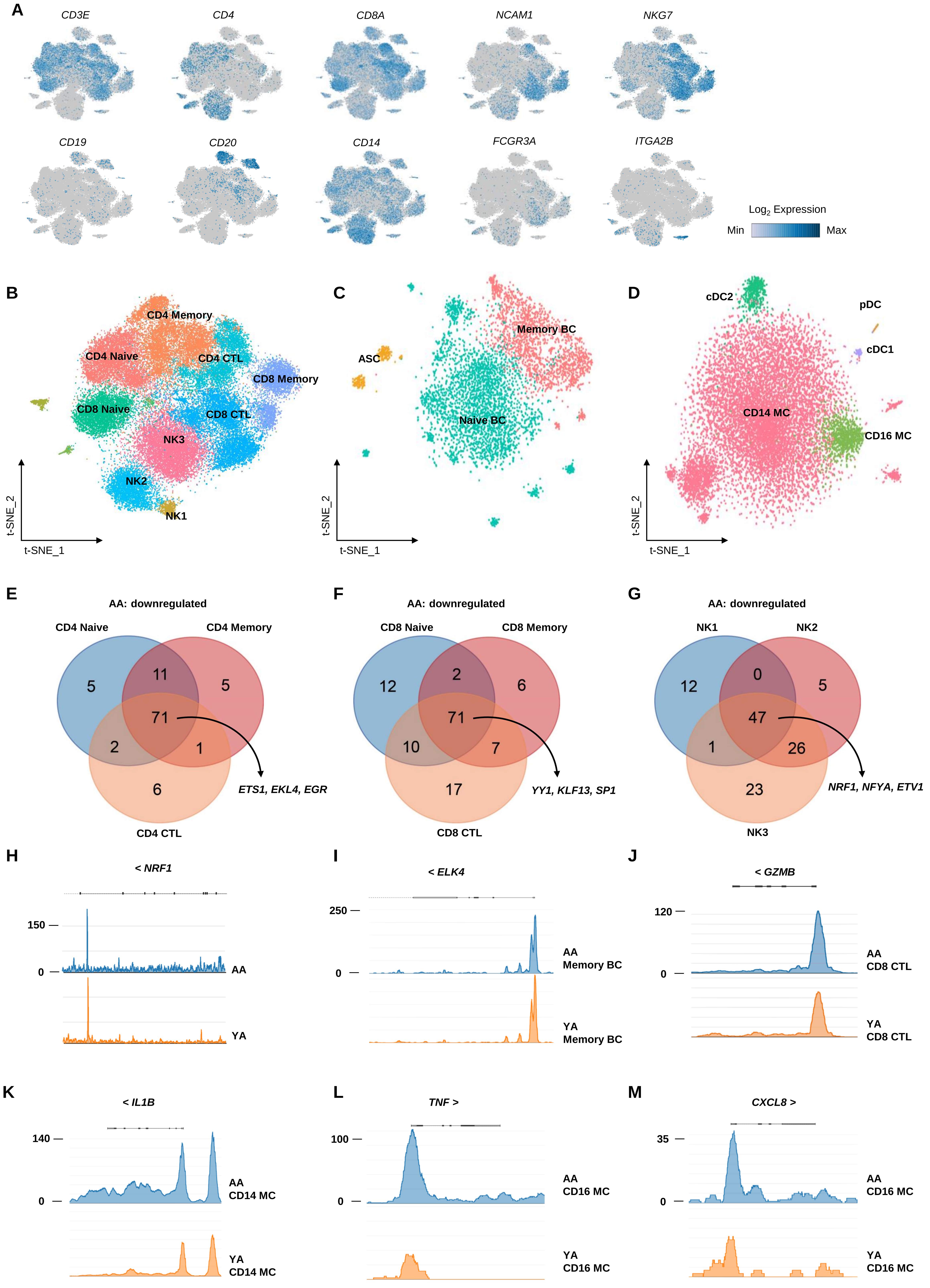

A
CD3E
CD4
CD8A
NCAM1
NKG7
CD19
CD20
CD14
FCGR3A
ITGA2B
Log2 Expression
Min
Max
B
CD4 Memory
CD4 Naive
CD4 CTL
CD8 Memory
CD8 Naive
CD8 CTL
NK3
NK2
NK1
t-SNE_2
t-SNE_1
C
ASC
Memory BC
Naive BC
t-SNE_2
t-SNE_1
D
cDC2
pDC
cDC1
CD14 MC
CD16 MC
t-SNE_2
t-SNE_1
E
AA: downregulated
CD4 Naive
CD4 Memory
5
11
5
71
2
1
6
ETS1, EKL4, EGR
CD4 CTL
F
AA: downregulated
CD8 Naive
CD8 Memory
12
2
6
71
10
7
17
YY1, KLF13, SP1
CD8 CTL
G
AA: downregulated
NK1
NK2
12
0
5
47
1
26
23
NRF1, NFYA, ETV1
NK3
H
< NRF1
150
0
AA
YA
I
< ELK4
250
0
AA
Memory BC
YA
Memory BC
J
< GZMB
120
0
AA
CD8 CTL
YA
CD8 CTL
K
< IL1B
140
0
AA
CD14 MC
YA
CD14 MC
L
TNF >
100
0
AA
CD16 MC
YA
CD16 MC
M
CXCL8 >
35
0
AA
CD16 MC
YA
CD16 MC

Figure. S9

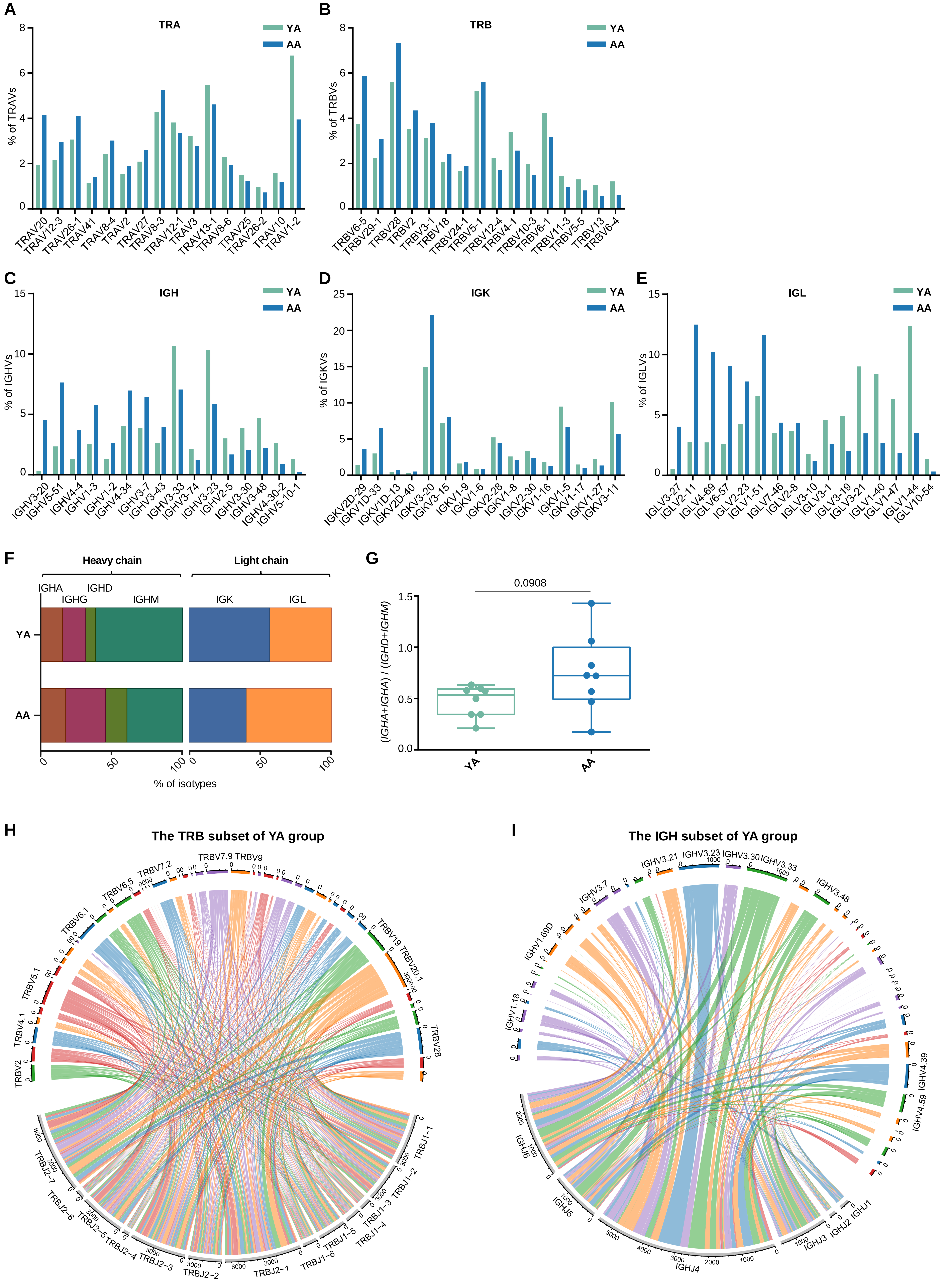

A
TRA
YA
AA
% of TRAVs
TRAV20 TRAV12-3 TRAV26-1 TRAV41 TRAV8-4 TRAV2 TRAV27 TRAV8-3 TRAV12-1 TRAV3 TRAV13-1 TRAV8-6 TRAV25 TRAV26-2 TRAV10 TRAV1-2
B
TRB
% of TRBVs
TRBV6-5 TRBV29-1 TRBV28 TRBV2 TRBV3-1 TRBV18 TRBV24-1 TRBV5-1 TRBV12-4 TRBV4-1 TRBV10-3 TRBV6-1 TRBV11-3 TRBV5-5 TRBV13 TRBV6-4
C
IGH
% of IGHVs
IGHV3-20 IGHV5-51 IGHV4-4 IGHV1-3 IGHV1-2 IGHV4-34 IGHV3-7 IGHV3-43 IGHV3-33 IGHV3-74 IGHV3-23 IGHV2-5 IGHV3-30 IGHV3-48 IGHV4-30-2 IGHV5-10-1
D
IGK
% of IGKVs
IGKV2D-29 IGKV1D-33 IGKV1D-13 IGKV2D-40 IGKV3-20 IGKV3-15 IGKV1-9 IGKV1-6 IGKV2-28 IGKV1-8 IGKV2-30 IGKV1-16 IGKV1-5 IGKV1-17 IGKV1-27 IGKV3-11
E
IGL
% of IGLVs
IGLV3-27 IGLV2-11 IGLV4-69 IGLV6-57 IGLV2-23 IGLV1-51 IGLV7-46 IGLV2-8 IGLV3-10 IGLV3-1 IGLV3-19 IGLV3-21 IGLV1-40 IGLV1-47 IGLV1-44 IGLV10-54
F
Heavy chain
Light chain
IGHA IGHG IGHD IGHM
IGK IGL
% of isotypes
G
0.0908
(IGHA+IGHA) / (IGHD+IGHM)
H
The TRB subset of YA group
I
The IGH subset of YA group

Figure. S10

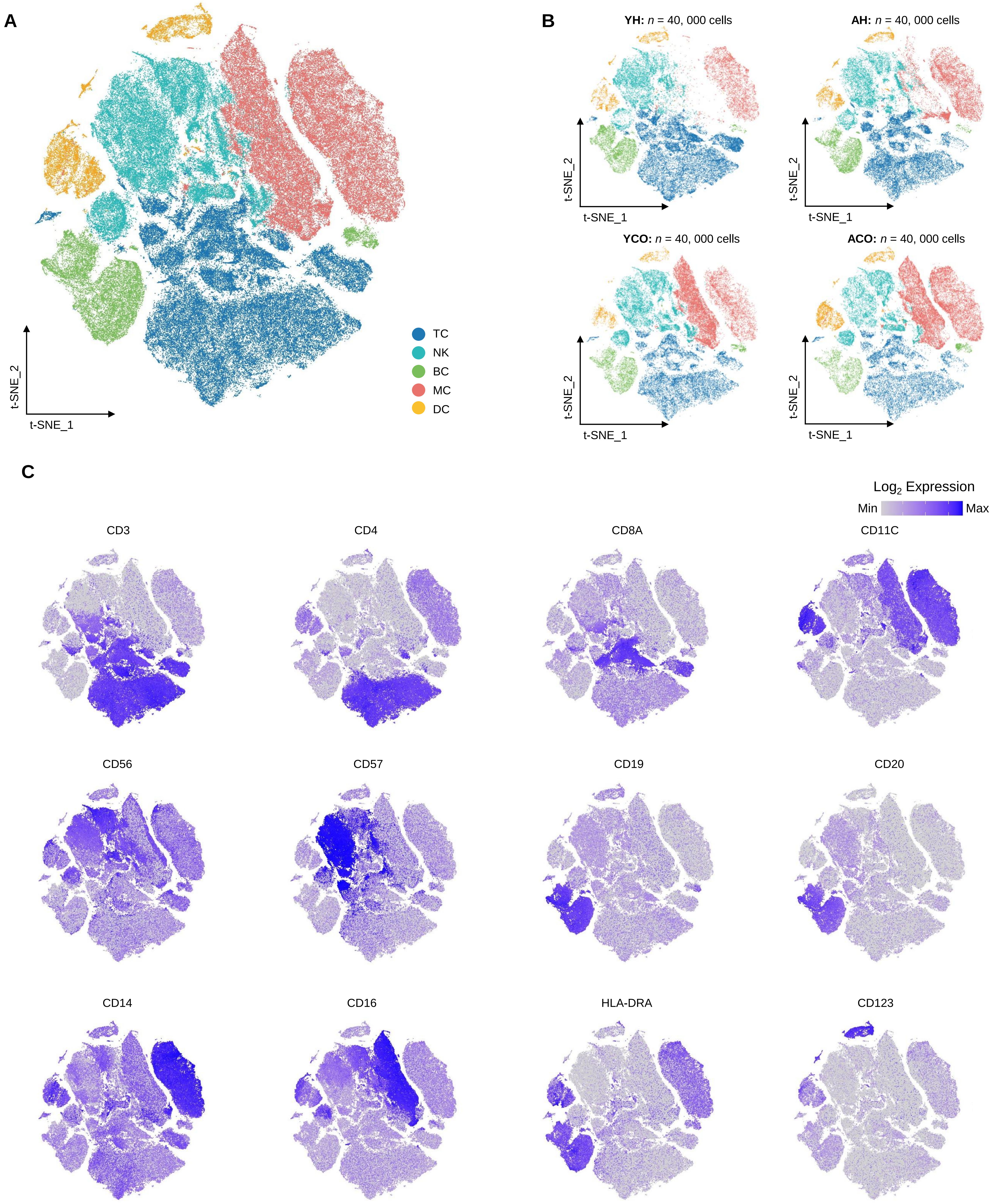
A
t-SNE_2
t-SNE_1
TC
NK
BC
MC
DC
B
YH: n = 40, 000 cells
AH: n = 40, 000 cells
YCO: n = 40, 000 cells
ACO: n = 40, 000 cells
t-SNE_2
t-SNE_1
C
Log2 Expression
Min
Max
CD3
CD4
CD8A
CD11C
CD56
CD57
CD19
CD20
CD14
CD16
HLA-DRA
CD123

Figure. S11

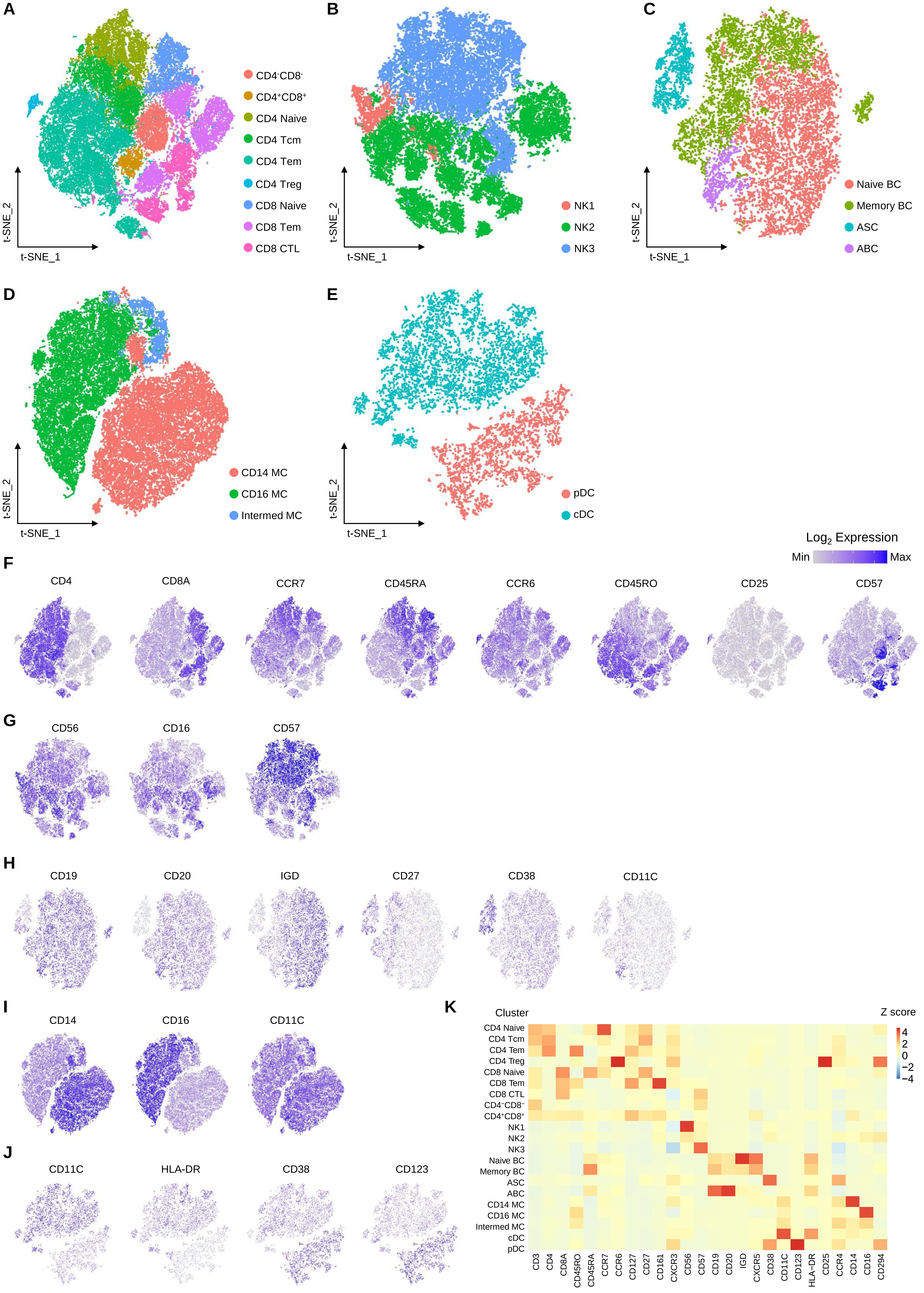

A
CD4⁻CD8⁻
CD4⁺CD8⁺
CD4 Naive
CD4 Tcm
CD4 Tem
CD4 Treg
CD8 Naive
CD8 Tem
CD8 CTL
t-SNE_2
t-SNE_1
B
NK1
NK2
NK3
C
Naive BC
Memory BC
ASC
ABC
D
CD14 MC
CD16 MC
Intermed MC
E
pDC
cDC
Log2 Expression
Min
Max
F
CD4
CD8A
CCR7
CD45RA
CCR6
CD45RO
CD25
CD57
G
CD56
CD16
CD57
H
CD19
CD20
IGD
CD27
CD38
CD11C
I
CD14
CD16
CD11C
J
CD11C
HLA-DR
CD38
CD123
K
Cluster
Z score
4
2
0
−2
−4
CD4 Naive
CD4 Tcm
CD4 Tem
CD4 Treg
CD8 Naive
CD8 Tem
CD8 CTL
CD4⁻CD8⁻
CD4⁺CD8⁺
NK1
NK2
NK3
Naive BC
Memory BC
ASC
ABC
CD14 MC
CD16 MC
Intermed MC
cDC
pDC
CD3
CD4
CD8A
CD45RO
CD45RA
CCR7
CCR6
CD127
CD27
CD161
CXCR3
CD56
CD57
CD19
CD20
IGD
CXCR5
CD38
CD11C
CD123
HLA-DR
CD25
CCR4
CD14
CD16
CD294

Figure. S12

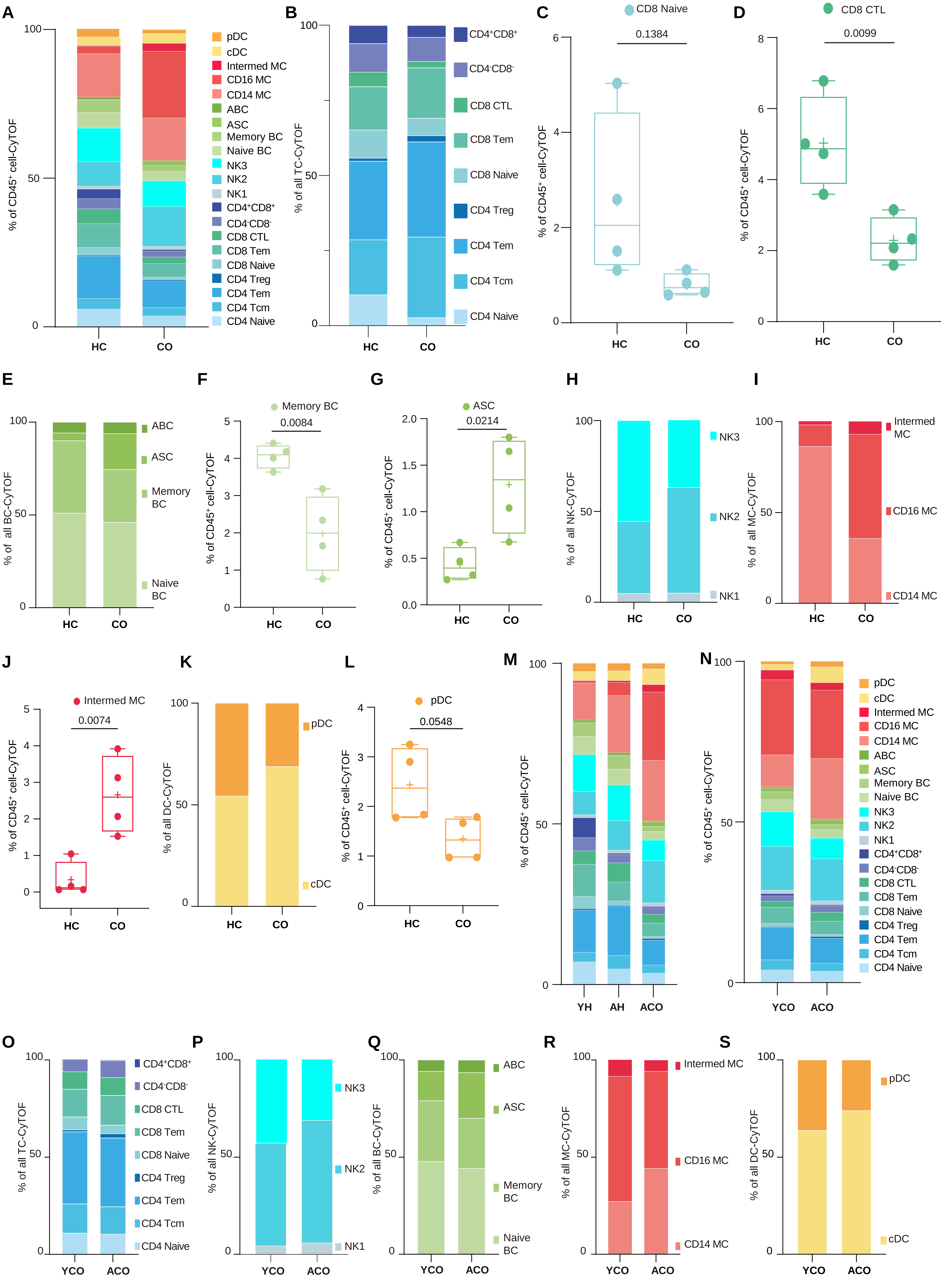
A
B
C
D
E
F
G
H
I
J
K
L
M
N
O
P
Q
R
S
% of CD45+ cell-CyTOF
% of all TC-CyTOF
% of all BC-CyTOF
% of all NK-CyTOF
% of all MC-CyTOF
% of all DC-CyTOF
pDC
cDC
Intermed MC
CD16 MC
CD14 MC
ABC
ASC
Memory BC
Naive BC
NK3
NK2
NK1
CD4+CD8+
CD4-CD8-
CD8 CTL
CD8 Tem
CD8 Naive
CD4 Treg
CD4 Tem
CD4 Tcm
CD4 Naive
0.1384
0.0099
0.0084
0.0214
0.0074
0.0548
HC
CO
YH
AH
ACO
YCO

Figure. S13

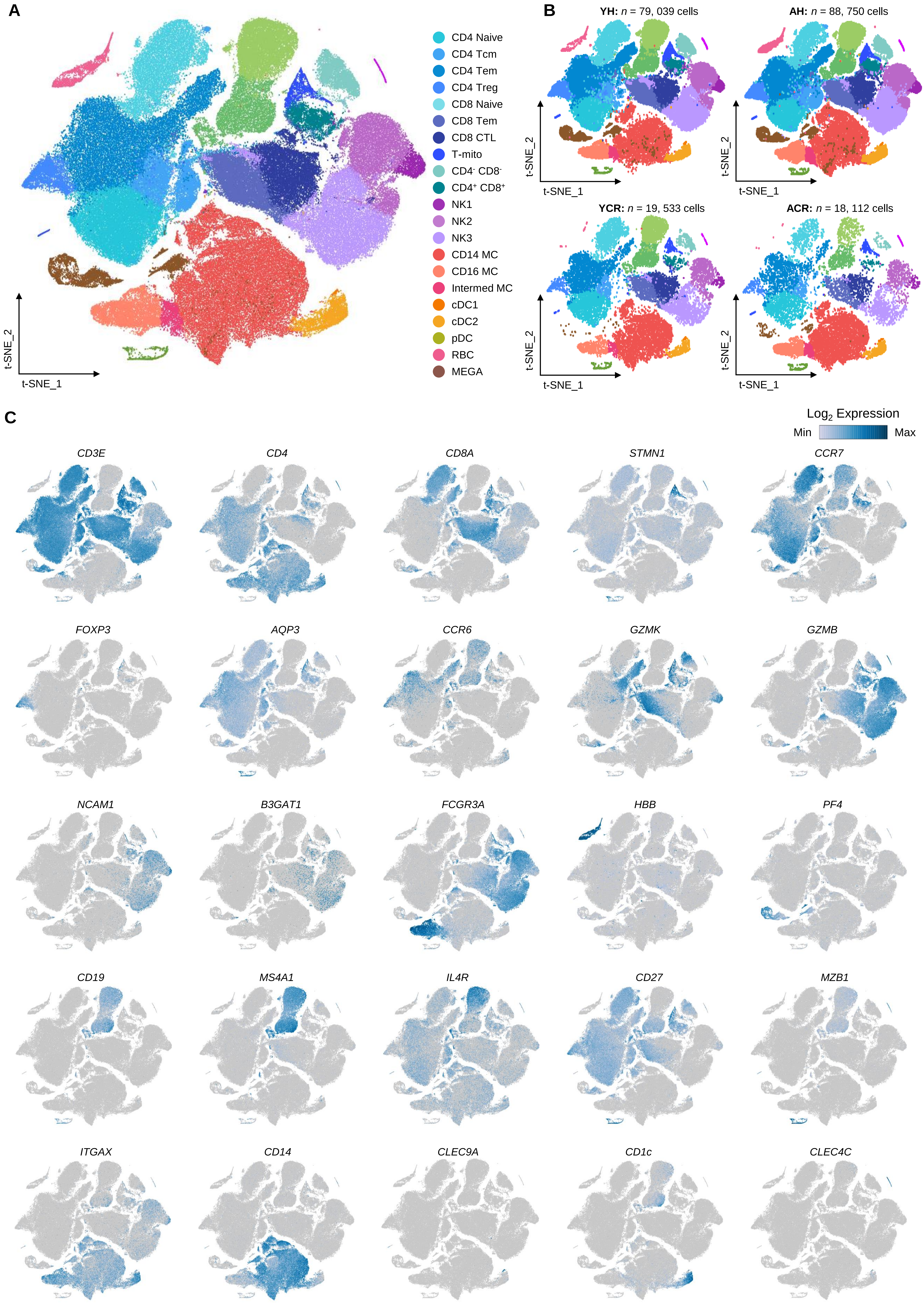
A
CD4 Naive
CD4 Tcm
CD4 Tem
CD4 Treg
CD8 Naive
CD8 Tem
CD8 CTL
T-mito
CD4- CD8-
CD4+ CD8+
NK1
NK2
NK3
CD14 MC
CD16 MC
Intermed MC
cDC1
cDC2
pDC
RBC
MEGA
t-SNE_1
t-SNE_2
B
YH: n = 79, 039 cells
AH: n = 88, 750 cells
YCR: n = 19, 533 cells
ACR: n = 18, 112 cells
C
Log2 Expression
Min
Max
CD3E
CD4
CD8A
STMN1
CCR7
FOXP3
AQP3
CCR6
GZMK
GZMB
NCAM1
B3GAT1
FCGR3A
HBB
PF4
CD19
MS4A1
IL4R
CD27
MZB1
ITGAX
CD14
CLEC9A
CD1c
CLEC4C

Figure. S14

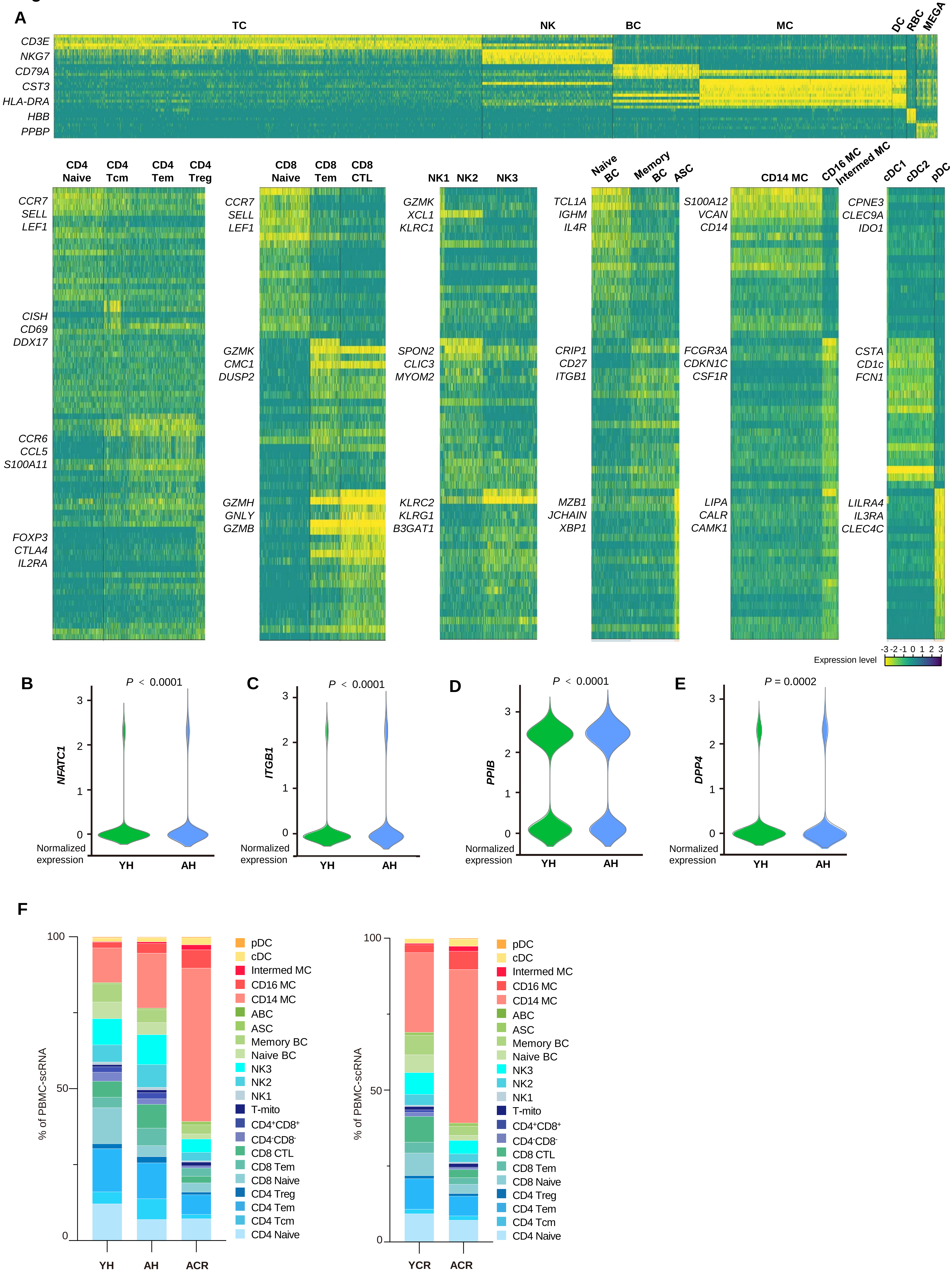
A
TC
NK
BC
MC
DC
RBC
MEGA
CD3E
NKG7
CD79A
CST3
HLA-DRA
HBB
PPBP
CD4 Naive
CD4 Tcm
CD4 Tem
CD4 Treg
CCR7
SELL
LEF1
CISH
CD69
DDX17
CCR6
CCL5
S100A11
FOXP3
CTLA4
IL2RA
CD8 Naive
CD8 Tem
CD8 CTL
GZMK
CMC1
DUSP2
GZMH
GNLY
GZMB
NK1
NK2
NK3
XCL1
KLRC1
SPON2
CLIC3
MYOM2
KLRC2
KLRG1
B3GAT1
Naive BC
Memory BC
ASC
TCL1A
IGHM
IL4R
CRIP1
CD27
ITGB1
MZB1
JCHAIN
XBP1
CD14 MC
CD16 MC
Intermed MC
S100A12
VCAN
CD14
FCGR3A
CDKN1C
CSF1R
LIPA
CALR
CAMK1
cDC1
cDC2
pDC
CPNE3
CLEC9A
IDO1
CSTA
CD1c
FCN1
LILRA4
IL3RA
CLEC4C
Expression level
-3 -2 -1 0 1 2 3
B
P < 0.0001
NFATC1
Normalized expression
YH
AH
C
P < 0.0001
ITGB1
D
P < 0.0001
PPIB
E
P = 0.0002
DPP4
F
% of PBMC-scRNA
100
50
0
YH
AH
ACR
YCR
pDC
cDC
Intermed MC
CD16 MC
CD14 MC
ABC
ASC
Memory BC
Naive BC
NK3
NK2
NK1
T-mito
CD4+CD8+
CD4-CD8-
CD8 CTL
CD8 Tem
CD8 Naive
CD4 Treg
CD4 Tem
CD4 Tcm
CD4 Naive

Figure. S15

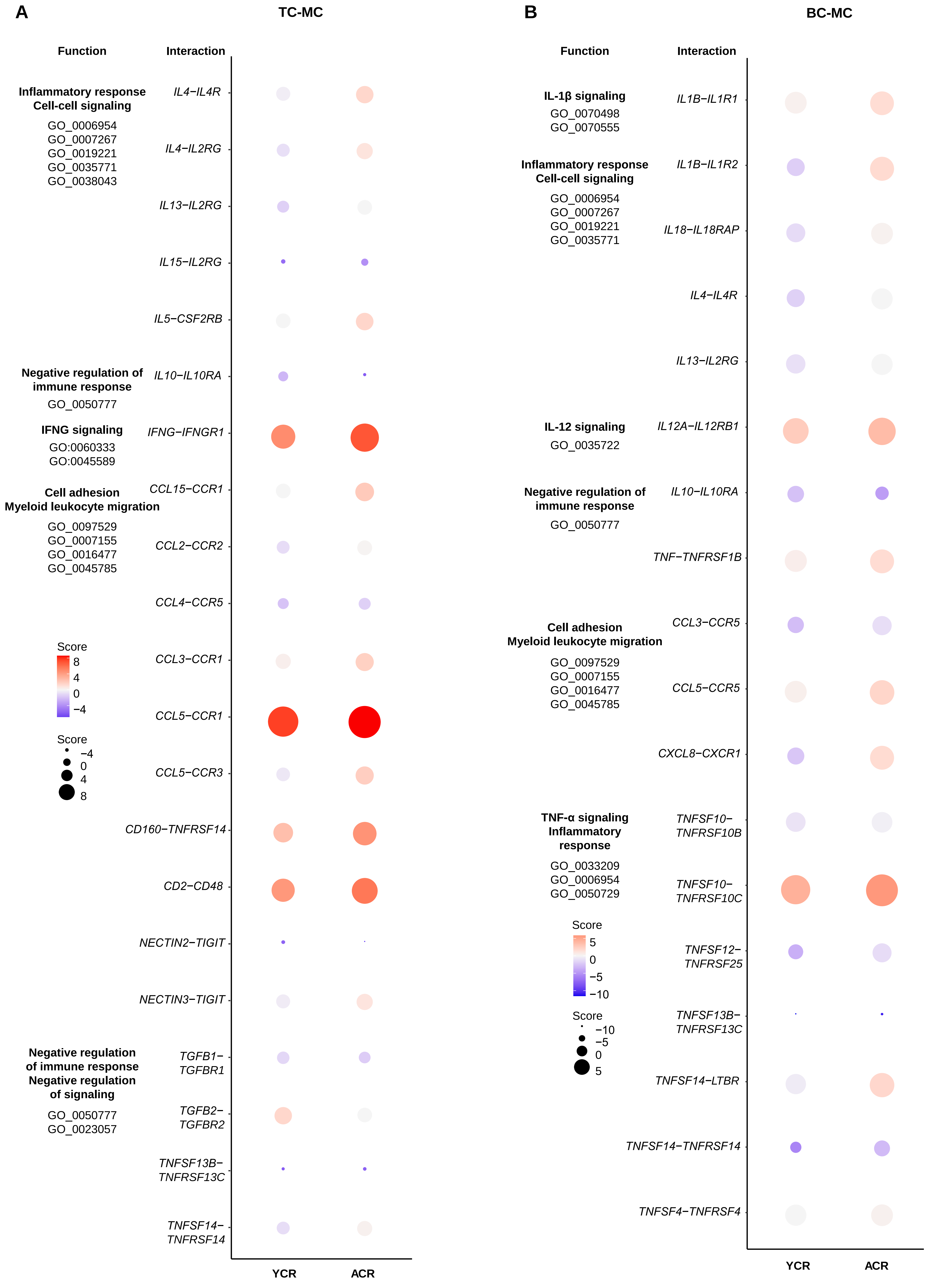
A
TC-MC
Function
Interaction
Inflammatory response
Cell-cell signaling
GO_0006954
GO_0007267
GO_0019221
GO_0035771
GO_0038043
IL4−IL4R
IL4−IL2RG
IL13−IL2RG
IL15−IL2RG
IL5−CSF2RB
Negative regulation of immune response
GO_0050777
IL10−IL10RA
IFNG signaling
GO:0060333
GO:0045589
IFNG−IFNGR1
Cell adhesion
Myeloid leukocyte migration
GO_0097529
GO_0007155
GO_0016477
GO_0045785
CCL15−CCR1
CCL2−CCR2
CCL4−CCR5
CCL3−CCR1
CCL5−CCR1
CCL5−CCR3
CD160−TNFRSF14
CD2−CD48
NECTIN2−TIGIT
NECTIN3−TIGIT
Negative regulation of immune response
Negative regulation of signaling
GO_0050777
GO_0023057
TGFB1−TGFBR1
TGFB2−TGFBR2
TNFSF13B−TNFRSF13C
TNFSF14−TNFRSF14
Score
8
4
0
−4
Score
−4
0
4
8
YCR
ACR
B
BC-MC
Function
Interaction
IL-1β signaling
GO_0070498
GO_0070555
IL1B−IL1R1
Inflammatory response
Cell-cell signaling
GO_0006954
GO_0007267
GO_0019221
GO_0035771
IL1B−IL1R2
IL18−IL18RAP
IL4−IL4R
IL13−IL2RG
IL-12 signaling
GO_0035722
IL12A−IL12RB1
Negative regulation of immune response
GO_0050777
IL10−IL10RA
TNF−TNFRSF1B
Cell adhesion
Myeloid leukocyte migration
GO_0097529
GO_0007155
GO_0016477
GO_0045785
CCL3−CCR5
CCL5−CCR5
CXCL8−CXCR1
TNF-α signaling
Inflammatory response
GO_0033209
GO_0006954
GO_0050729
TNFSF10−TNFRSF10B
TNFSF10−TNFRSF10C
TNFSF12−TNFRSF25
TNFSF13B−TNFRSF13C
TNFSF14−LTBR
TNFSF14−TNFRSF14
TNFSF4−TNFRSF4
Score
5
0
−5
−10
Score
−10
−5
0
5
YCR
ACR
